# Comparison of the effects of stent-based diversion technique versus prophylactic double-lumen ileostomy on intestinal flora in postoperative patients with rectal cancer

**DOI:** 10.3389/fmicb.2026.1791364

**Published:** 2026-07-21

**Authors:** Huimin Chen, Bo Zhang, Bingqing Zhu, Pengyang Zhou, Chao Xu, Qiken Li, Weiping Chen

**Affiliations:** 1 Graduate School of Wenzhou Medical University, Wenzhou, Zhejiang, China; 2 Department of Colorectal Surgery, Zhejiang Cancer Hospital, Hangzhou, Zhejiang, China; 3 Department of Medical Oncology, The Xin Hua Hospital of Zhejiang Province, Hangzhou, Zhejiang, China

**Keywords:** gut microbiota, metagenomic sequencing, mid-low rectal cancer, stent-based diversion technique, prophylactic double-lumen ileostomy

## Abstract

**Background:**

The stent-based diversion technique (SDT), as a novel surgical approach for reducing anastomotic leakage (AL) following low anterior resection (LAR), achieving effective intestinal diversion while avoiding ileostomy and subsequent stoma reversal surgery. Although multicenter randomized controlled trials have demonstrated the safety of SDT, the alterations in postoperative intestinal microbiota following SDT remain inadequately characterized.

**Methods:**

This study enrolled 40 patients with mid-low rectal cancer (21 SDT, 19 PDI). Rectal swab samples were collected preoperatively and at 3 weeks and 3 months postoperatively (*n* = 120) for metagenomic sequencing. α- and β-diversity analyses were performed to compare microbial community characteristics. LEfSe was used for differential analysis of species and KEGG functional pathways. Postoperative clinical outcomes including AL and anastomotic stricture (AS) were assessed.

**Results:**

The SDT group showed a significantly lower incidence of AS compared with the PDI group (4.76% vs. 31.58%, *p* < 0.05). Preoperative α- and β-diversity were comparable between groups. Postoperatively, the SDT group exhibited higher microbial richness at both 3 weeks and 3 months (both *p* < 0.05). In the PDI group, the α-diversity showed a continuous decline from 3 weeks to 3 months postoperatively compared with the preoperative baseline (*p* < 0.05). However, the SDT group demonstrated no significant decrease in α-diversity at 3 weeks (*p* > 0.05), but did at 3 months (*p* < 0.05). Significant intergroup β-diversity divergence emerged from 3 weeks onward (both *p* < 0.05). The SDT group showed significant structural changes from 3 weeks to 3 months (*p* < 0.05), whereas the PDI group remained stable. At 3 weeks, opportunistic pathogens (e.g., *Parvimonas micra*) were enriched in the PDI group, while the SDT group enriched beneficial taxa (e.g., *Akkermansia*). By 3 months, the PDI group exhibited enrichment of oral/genitourinary-derived bacteria (*Prevotellaceae*, *Porphyromonas*, *Fusobacterium*), whereas the SDT group showed higher abundance of beneficial Bacteroidota (e.g., *Phocaeicola vulgatus*). Functionally, the SDT group enriched amino acid and carbohydrate metabolism pathways, while the PDI group enriched translation and energy metabolism pathways.

**Conclusion:**

We found that SDT better preserves postoperative gut microbiota diversity, promotes the restoration of beneficial bacteria, and influences microbial functional pathways, thereby establishing a more favorable microbiome environment for patients.

## 1 Introduction

According to the 2022 global cancer statistics, colorectal cancer (CRC) ranks as the third most common malignancy and the second leading cause of cancer-related mortality worldwide (Bray et al., 2024). The 2025 Chinese Colorectal Cancer Surgery Registry Database reported that rectal cancer (50.2%) marginally exceeded colon cancer (49.8%) among surgical patients, with mid-to-low rectal cancer accounting for 70.9% of all rectal malignancies (Li et al., 2026). For curable mid-to-low rectal cancers, LAR is commonly employed as the radical treatment modality. However, AL, occurring in 1.5–20% of cases (Degiuli et al., 2022; Bekki et al., 2023; Rutegård et al., 2024), represents one of the most severe postoperative complications, potentially causing intra-abdominal sepsis, delayed recovery, impaired quality of life, and increased mortality. PDI is commonly employed for fecal diversion to reduce leakage risk (Albulescu et al., 2023), yet necessitates stoma reversal surgery 3–6 months postoperatively, with attendant risks of stoma-related complications including parastomal hernia, prolapse, retraction, and ischemic necrosis (Babakhanlou et al., 2022; Kumano et al., 2023; Hinojosa-Gonzalez et al., 2024). Additionally, reversal surgery carries procedural risks, increases healthcare costs, and imposes physiological, psychological, and socioeconomic burdens on patients (Colorectal Surgery Group of the Chinese Society of Surgery et al., 2025).

To address these limitations, SDT was developed as an innovative alternative that maintains effective fecal diversion while circumventing the drawbacks of PDI (Song et al., 2022; Tong et al., 2024). The procedure generally involves placement of a biodegradable double-ended stent into the intestinal lumen following LAR, stent fixation, and insertion of a mushroom-shaped drainage tube through an abdominal stoma proximal to the stent for postoperative intestinal content diversion. Around 3–4 weeks postoperatively, an abdominal X-ray is taken to assess stent degradation, after which the drainage tube is removed (Song et al., 2022; Tong et al., 2024). This approach avoids stoma-related morbidity, reduces postoperative care duration, decreases healthcare costs, and improves quality of life. Our group participated in a phase II clinical trial that evaluated SDT safety following LAR, and enrolled 80 patients (SDT: 37; Ileostomy: 43). The SDT group demonstrated 1 AL and no AS, compared with 3 AL and 2 AS in the PDI group, with significantly lower 90-day major complication rates (Clavien-Dindo ≥III) (Tong et al., 2024). AS, occurring in 20–30% of cases (Placer et al., 2010), represents another critical complication that may cause intestinal obstruction, delayed or failed stoma reversal, and permanent stoma dependency. Beyond technical factors such as inadequate blood supply, both AL and AS are closely associated with impaired anastomotic healing resulting from dysregulated intestinal inflammation (Dong et al., 2025; Yavuz et al., 2025). Early postoperative inflammation (3–4 days) is essential for bacterial clearance and initiation of tissue repair; however, excessive or prolonged inflammation promotes neutrophil overrecruitment, extracellular trap release, and sustained proinflammatory cytokine elevation (IL-1β, IL-6, TNF-α), thereby suppressing collagen deposition and angiogenesis required for healing. Intestinal microbiota plays a pivotal role in modulating this inflammatory process (Ma et al., 2025; Sun et al., 2026).

Growing evidence indicates that intestinal microbiota critically maintains gut barrier integrity, modulates local immunity, and promotes tissue repair, thereby influencing postoperative anastomotic healing and functional recovery in CRC patients (Hajjar et al., 2023). Preoperative antibiotic prophylaxis, bowel preparation, and altered intestinal anatomy following surgery induce substantial microbiota perturbations, characterized by depletion of obligate anaerobes (e.g., *Bifidobacterium*, *Turicibacter*, *Prevotella*) and expansion of facultative anaerobes (Enterobacteriaceae, *Enterococcus*, *Staphylococcus*) and aerobes (*Pseudomonas*) (Ohigashi et al., 2013; Kong et al., 2019). Such dysbiosis, with reduced beneficial and increased pathogenic taxa, compromises epithelial barrier function and elevates AL risk (Hajjar et al., 2023). Hajjar et al. (2023) identified two bacterial strains closely associated with anastomotic healing—*Alistipes onderdonkii* kh33 and *Parabacteroides goldsteinii* kh35—by comparing preoperative microbiota between patients with and without leakage. These strains exacerbate leakage by upregulating mucosal proinflammatory cytokines (MIP-1α, MIP-2, MCP-1, IL-17A/F). Jin et al. (2022) demonstrated via 16S rRNA sequencing that non-leakage (nCAL) and leakage (CAL) patients harbored distinct microbiota profiles. Fecal microbiota transplantation from nCAL donors accelerated colonic anastomotic healing in rats via TGF-β/Smad pathway activation, induction of epithelial-mesenchymal transition, and enhanced collagen synthesis. Additionally, *Lactobacillus reuteri* promotes wound healing by generating lactate to activate the oxytocin signaling pathway and vagal afferents, or by stimulating secretin-mediated oxytocin release from intestinal epithelial cells to activate CD4+Foxp3+CD25+T cells (Poutahidis et al., 2013; Danhof et al., 2023). Collectively, these findings establish intestinal microbiota as a critical modulator of postoperative recovery, particularly anastomotic healing, in CRC surgery.

Despite the established clinical efficacy of both approaches, the differential impact of SDT and PDI on postoperative intestinal microbiota remains poorly understood. This study collected rectal swab samples at three time points (1 day preoperatively, 3 weeks and 3 months postoperatively) from patients who underwent either procedure, and employed metagenomic sequencing to characterize the dynamic trajectories of gut microbiota from three perspectives: diversity, taxonomic composition, and KEGG functional pathways. This study provides direct evidence clarifying the distinct influences of these two diversion strategies on intestinal microecology, and offer novel insights into optimizing perioperative gut management and developing microbiota-targeted strategies for complication prevention.

## 2 Materials and methods

### 2.1 Study participants

This study was approved by the Ethics Committee of Zhejiang Cancer Hospital (Approval number: IRB-2024-694), and conducted in accordance with the Declaration of Helsinki. Patient information was prospectively reviewed, and we recruited individuals with rectal cancer diagnosed at Zhejiang Cancer Hospital between June 2024 and July 2025. Eligible participants were patients aged 18–80 years with an ECOG performance status ≤1 and an ASA physical status ≤III, who were diagnosed with primary rectal cancer and assessed as high-risk for AL following laparoscopic radical resection. High-risk status was defined by the presence of at least one of the following criteria: BMI ≥ 30 kg/m^2^, long-term preoperative corticosteroid use, poor preoperative general condition (e.g., albumin <30.0 g/L, renal failure requiring renal replacement therapy, or diabetes mellitus), receipt of neoadjuvant radiotherapy, tumor distance from the anal verge ≤7 cm, or the presence of intraoperative high-risk factors (e.g., use of ≥3 linear stapler firings, defects between staple lines, or a positive intraoperative air-leak test). Exclusion criteria included: severe cardiopulmonary disease, primary intestinal disorders (such as Crohn’s disease or ulcerative colitis), intestinal obstruction or intra-abdominal infection, use of medications known to impair anastomotic healing (e.g., corticosteroids or immunosuppressants), antibiotic use within 2 weeks prior to sampling, use of probiotics or microbiota-modulating agents within 1 week, and any other condition deemed unsuitable for participation by the investigators. Postoperative specimens were examined and diagnosed by pathological experts. Following stringent review, 40 patients were deemed eligible and enrolled in the study. All participants provided written informed consent prior to sample collection.

### 2.2 Study procedure and fecal sample collection

Eligible patients were randomized (1:1) to the SDT or PDI group using a computer-generated random number table created by SPSS software (version 26.0; IBM Corp.) with block sizes of 4 and 6, resulting in 21 patients who underwent anterior resection with SDT (study group), and 19 underwent anterior resection with PDI (control group). The surgical procedures for SDT and PDI in patients underwent LAR have been described in detail in our previous publication, “*Safety of the stent-based diverting technique after low anterior resection in patients with rectal cancer*” (Tong et al., 2024). Briefly, in the SDT group, a biodegradable occlusive stent was placed and fixed in the ileal lumen 30 cm proximal to the ileocecal valve to completely occlude the intestinal lumen after LAR. A mushroom-shaped drainage tube was then inserted into the ileal lumen 10 cm proximal to the occlusive stent to divert intestinal contents. The mushroom tube was exteriorized through the right lower abdominal wall, properly fixed, and connected to a negative-pressure suction bulb for continuous drainage. The stent spontaneously disintegrated 3–4 weeks post-surgery. In contrast, in the PDI group, a standard prophylactic double-lumen ileostomy was created approximately 15–20 cm proximal to the ileocecal valve, and stoma reversal was scheduled 3–6 months after the primary surgery.

Rectal content samples were collected using a rectal swab at three time points: 1 day before surgery (prior to mechanical bowel preparation), 3 weeks postoperatively, and 3 months postoperatively (for patients in the PDI group, samples were collected before mechanical bowel preparation for stoma reversal). The procedure was performed as follows: a sterile cotton swab was inserted through the anus approximately 5–7 cm into the rectum and rotated against the rectal wall to obtain a mixed fecal-mucosal sample. The swab was then immediately placed into a sterile tube and stored at −80 °C. This method serves as an alternative to fecal collection in the Human Microbiome Project (HMP) protocol (Bansal et al., 2018).

### 2.3 DNA extraction and metagenomic sequencing

Total DNA was isolated from rectal swab samples using a MagPure Soil DNA LQ Kit (Magen, China) following the manufacturer’s instructions. DNA concentration and integrity were assessed by a NanoDrop 2000 spectrophotometer (Thermo Fisher Scientific, Waltham, MA, USA) and agarose gel electrophoresis, respectively. DNA was fragmented by S220 Focused-ultrasonicator (Covaris, USA) and cleaned up by Agencourt AMPure XP beads (Beckman Coulter Co., USA). Then the libraries were constructed using VAHTS Universal Plus DNA Library Prep Kit for Illumina® (Vazyme，China) according to the manufacturer’s instructions. The metagenome sequencing and analysis were conducted by OE Biotech Co., Ltd. (Shanghai, China).

### 2.4 Bioinformatic analysis

The libraries were sequenced on an Illumina Novaseq 6000 platform and 150 bp paired-end reads were generated. Sequences in the FastQ file were trimmed and filtered using fastp (v 0.20.1). Host DNA decontamination was performed. The post-filtered pair-end reads were aligned against the host genome using BBMap (v 38.93-0) and the aligned reads were discarded. Metagenome assembly was performed using MEGAHIT (v 1.2.9) after getting valid reads. Use gaps inside scaffold as breakpoint to interrupt the scaffold into new contigs (scaftig), and these new Scaftigs with length >500 bp were retained. ORF prediction was performed on assembled scaffolds using Prodigal (v2.6.3) was performed and translated into amino acid sequences. The non-redundant gene sets were built for all predicted genes using MMSeqs2 (v 13.45111). The clustering parameters were 95% identity and 90% coverage. The longest gene was selected as the representative sequence of each gene set. Clean reads of each sample were aligned against the non-redundant gene set (95% identity) use Salmon (v 1.8.0), and the abundance information of the gene in the corresponding sample was counted. The taxonomy of the species was obtained as a result of the corresponding taxonomy database of the NR Library, and the abundance of the species was calculated using the corresponding abundance of the genes. To construct abundance profiles at each taxonomic level, abundance statistics were performed at each level of Domain, Kingdom, Phylum, Class, Order, Family, Genus and Species. The gene set representative sequence (amino acid sequence) was annotated with NR, KEGG, eggNOG, SWISSPROT and GO database with an *e*-value of 1*e*−5 using DIAMOND (v0.9.10.111).

#### 2.4.1 Microbiota diversity analysis

α-diversity was evaluated using the Chao1, observed species (Obs), Shannon, and Simpson indices based on species abundance profiles. β-diversity was assessed by principal coordinate analysis (PCoA) using Bray–Curtis dissimilarity metrics. Intergroup differences were analyzed using the t-test or Wilcoxon rank-sum test, and linear discriminant analysis effect size (LEfSe) was employed to identify discriminative taxa. β-diversity differences between groups were evaluated using permutational multivariate analysis of variance (PERMANOVA; Adonis2 test with 999 permutations). Statistical significance was set at *p* < 0.05. Data analysis and visualization were conducted using R (version 4.1.2).

#### 2.4.2 Differential analysis of microbiota and KEGG functional pathways

To identify significant intergroup differences in species abundance, the Shapiro–Wilk test was first applied to assess normality for two-group comparisons. Welch’s *t*-test was used for normally distributed data with homogeneity of variance, whereas the Wilcoxon rank-sum test was employed for non-normally distributed or heteroscedastic data. For comparisons involving three or more independent groups, the Kruskal–Wallis test was applied, followed by Dunn’s post-hoc test for pairwise comparisons when appropriate. Statistical significance was defined as *p* < 0.05. Significantly different functions were selected based on *p* values, and the top 10 most abundant features were visualized using boxplots. Additionally, LEfSe analysis was performed to identify characteristic species and KEGG functional pathways with significant intergroup differences (LDA score > 2.0, *p* < 0.05), with results presented as cladograms and LDA score bar charts. All visualizations were generated using R software (version 4.1.2).

### 2.5 Statistical analysis

Normality of continuous variables was assessed using the Shapiro–Wilk test. Normally distributed data were expressed as mean (standard deviation), whereas non-normally distributed data were presented as median (interquartile range). Categorical variables were described as frequency (percentage). For intergroup comparisons, Welch’s *t*-test or analysis of variance (ANOVA) was used for normally distributed continuous variables in two-group or multi-group comparisons, respectively; the Wilcoxon rank-sum test or Kruskal–Wallis test was employed for non-normally distributed variables. Categorical variables were compared using the *χ*^2^ test, Fisher’s exact test was used when the expected frequency was <5. All statistical analyses were performed using R software (version 4.1.2). Statistical significance was defined as *p* < 0.05.

## 3 Results

### 3.1 Baseline characteristics, perioperative outcomes, and anastomotic complications

Prior to metagenomic sequencing, baseline clinicopathological characteristics were compared between the SDT (*n* = 21) and PDI (*n* = 19) groups (Table 1). No statistically significant differences were observed between the two groups regarding baseline characteristics and tumor-related indicators. Postoperative outcomes and postoperative complications are shown in Table 2. The operative time was longer in the SDT group than in the PDI group (245 min vs. 204 min, *p* < 0.05). The total cost of the primary and secondary surgeries in the PDI group was significantly higher than that in the SDT group (RMB 80,000 vs. 51,000, *p* < 0.05). Regarding anastomotic complications, one case of AL occurred in each group (4.76% vs. 5.26%, *p* > 0.05). However, the incidence of AS (including membranous stricture) was significantly lower in the SDT group than in the PDI group (4.76% vs. 31.58%, *p* < 0.05). In the SDT group, one patient developed a grade II stricture that prevented passage of a 12-mm colonoscope, whereas six patients in the PDI group developed membranous strictures that were treatable with finger dilatation. The overall incidence of AS was 17.5%. One patient in the SDT group required a secondary diverting ileostomy surgery due to AL.

**Table 1:** Baseline demographic and clinical characteristics of study groups.

Characteristics	Group		*p* value^b^
	SDT group	PDI group	
	*N* = 21^a^	*N* = 19^a^	
Age (years)	61.90 ± 11.55	62.42 ± 11.22	0.885
Gender			0.141
Male	12 (57.1%)	15 (78.9%)	
Female	9 (42.9%)	4 (21.1%)	
BMI			0.904
<18.5	1 (4.76%)	1 (5.26%)	
18.5–24	9 (42.86%)	9 (47.37%)	
24–28	7 (33.33%)	7 (36.84%)	
≥28	4 (19.05%)	2 (10.53%)	
Distance of tumor from anal margin	6.30 (5.00, 7.00)	7.00 (6.50, 7.00)	0.284
Differentiation			0.113
Low	1 (4.76%)	2 (10.53%)	
Low-Middle	2 (9.52%)	6 (31.58%)	
Middle	13 (66.67%)	9 (47.37%)	
Middle-High	4 (19.05%)	2 (10.53%)	
High	1 (5.00%)	0 (0.00%)	
T stage			0.334
1	8 (38.10%)	5 (26.32%)	
2	5 (23.81%)	9 (47.37%)	
3	4 (19.05%)	4 (21.05%)	
4	4 (19.05%)	1 (5.26%)	
N stage			0.242
0	16 (76.19%)	13 (68.42%)	
1	3 (14.29%)	6 (31.58%)	
2	2 (9.52%)	0 (0.00%)	
Pathological staging			0.456
I	8 (38.10%)	4 (21.1%)	
II	8 (38.10%)	8 (42.1%)	
III	5 (23.81%)	7 (36.8%)	
IV	0 (0.00%)	0 (0.00%)	
Hypertension	5 (23.81%)	9 (47.37%)	0.119
Diabetes	0 (0.00%)	3 (15.79%)	0.098
History of abdominal surgery	5 (23.81%)	3 (15.79%)	0.698
Neoadjuvant therapy			0.664
Absent	17 (80.95%)	17 (89.47%)	
Present	4 (19.05%)	2 (10.53%)	
WBC (10^9^/L)	5.13 ± 1.59	5.99 ± 1.41	0.077
PLT (10^9^/L)	218 ± 69	246 ± 71	0.205
HB (g/L)	135 ± 13	139 ± 15	0.371
ALT (U/L)	14 (10, 24)	17 (12, 27)	0.455
AST (U/L)	20 (16, 31)	21 (16, 24)	0.737
ALB (U/L)	43.93 ± 3.14	44.15 ± 2.46	0.802
TB (μmol/L)	12.1 ± 5.2	11.0 ± 5.1	0.490
Cr (μmol/L)	57.90(42.8, 76.1)	68.30(48.9, 94.8)	0.083
CRP (mg/L)	1.88 (1.29, 4.54)	3.04 (1.98, 5.40)	0.333
PCT (ng/mL)	0.04 (0.02, 0.04)	0.04(0.03, 0.06)	0.155
CEA (ng/mL)	3 (2, 5)	3 (2, 7)	0.715
CA199 (U/mL)	15 (9, 20)	9 (7, 24)	0.533
INR	0.95 (0.92, 0.97)	0.96 (0.93, 1.01)	0.340
Tumor size (cm)	4.12 ± 1.47	3.82 ± 1.15	0.472
Anastomosis to anus (cm)	3.00 (2.00, 3.00)	3.00 (2.00, 4.00)	0.357
Postoperative adjuvant chemotherapy	12 (57.14%)	11 (57.89%)	0.962
Postoperative adjuvant radiotherapy	5 (23.81%)	2 (10.53%)	0.412

a Mean ± SD; Median (Q1, Q3); *n* (%).

b Welch Two Sample *t*-test; Wilcoxon rank sum test; Pearson’s Chi-squared test; Fisher’s exact test.

*p* < 0.05 was considered statistically significant. SDT, stent-based diverting technique; PDI, prophylactic double-lumen ileostomy; BMI, body mass index (calculated as weight in kilograms divided by height in meters squared); TNM stage was according to the American Joint Committee on Cancer (8th version). WBC, white blood cell; PLT, pancreolauryl test; HB, Hemoglobin; ALT, Alanine aminotransferase; AST, aspartate transaminase; ALB, Albumin; TB, total bilirubin; Cr, Serum Creatinine; CRP, C-reactive protein; PCT, Procalcitonin; CEA, carcinoembryonic antigen; CA 19-9, carbohydrate antigen 19-9; INR, international normalized ratio.

**Table 2:** Perioperative outcomes and postoperative complications.

Characteristics	Group		*p* value^b^
	SDT group	PDI group	
	*N* = 21^a^	*N* = 19^a^	
Operation time (min)	245 ± 63	204 ± 54	0.032
Anastomosis to anus (cm)	3.00 (2.00, 3.00)	3.00 (2.00, 4.00)	0.357
Time to abdominal drainage tube removal after primary surgery (days)	6 (6, 7)	6 (5, 7)	0.889
Time to initiation of liquid diet after primary surgery (days)	5 (5, 7)	4 (3, 7)	0.278
Length of hospital stay after primary surgery (days)	11 (9, 11)	8 (7, 11)	0.084
Hospitalization cost for primary surgery (×10,000 CNY)	5.10 (4.80, 5.90)	5.60 (4.80, 6.10)	0.424
Total hospitalization cost (×10,000 CNY)^c^	5.10 (4.80, 5.90)	8.00 (7.20, 8.80)	< 0.001
Abdominal complications	6 (28.57%)	4 (21.05%)	0.721
Anastomotic leakage	1 (4.76%)	1 (5.26%)	>0.999
Anastomotic stenosis	1 (4.76%)	6 (31.58%)	0.040

a Mean ± SD; Median (Q1, Q3); *n* (%).

b Welch Two Sample *t*-test; Wilcoxon rank sum test; Pearson’s Chi-squared test; Fisher’s exact test.

c This represents all hospitalization costs, with the stoma group including both the hospitalization costs during radical surgery and those for stoma closure surgery.

*p* < 0.05 was considered statistically significant. SDT, stent-based diverting technique; PDI, prophylactic double-lumen ileostomy; NA, not applicable.

### 3.2 Bacterial diversity analysis of SDT and PDI groups

**Figure 1:**
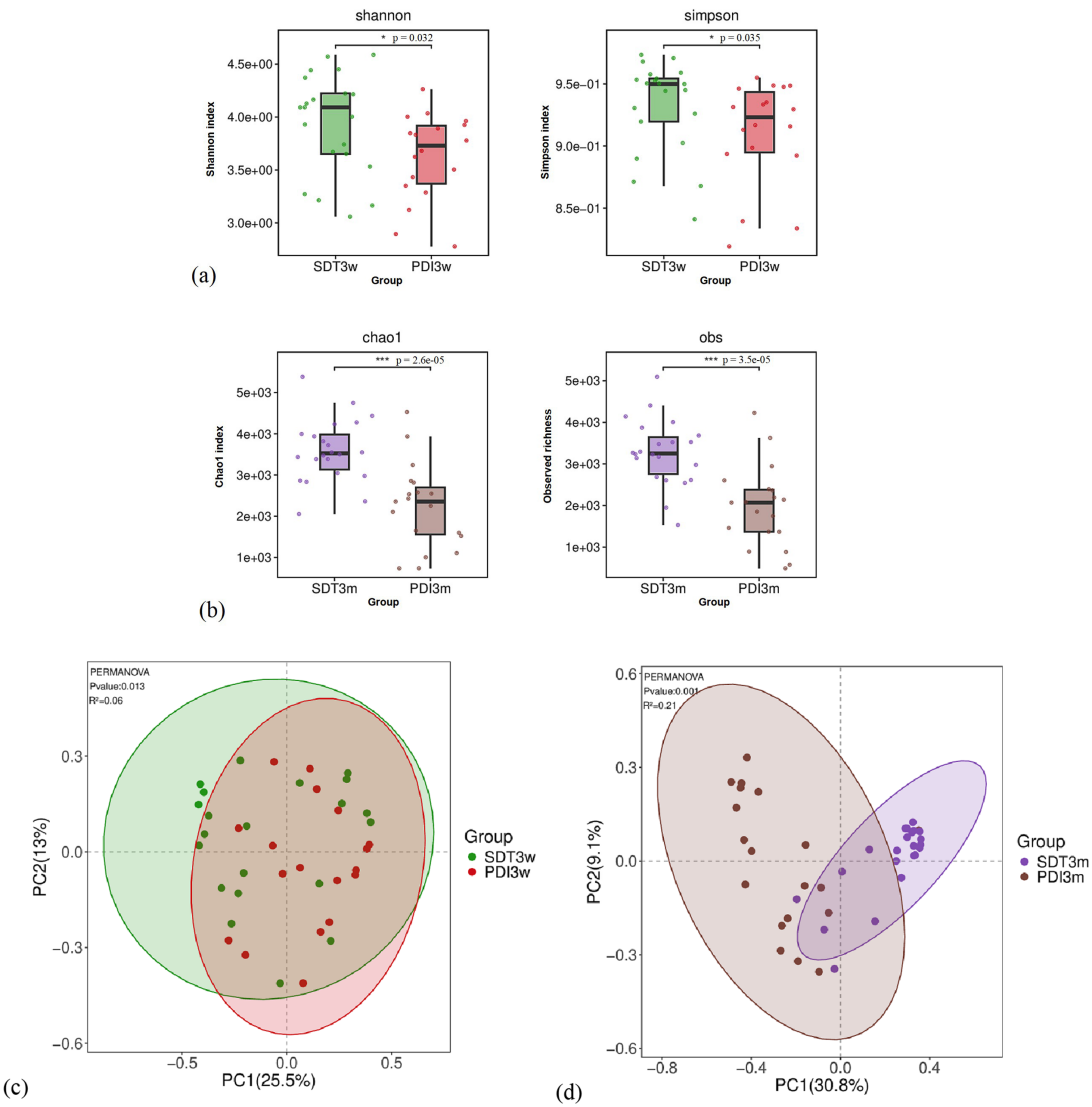
Comparison of gut microbiota α- and β-diversity between the SDT and PDI groups. **(a,b)** α-diversity indices (Simpson, Shannon, Obs, and Chao1) at 3 weeks and 3 months postoperatively. Box plots show the median, interquartile range, and data distribution. Significance levels are indicated above the comparisons (*p < 0.05, **p < 0.01, ***p < 0.001). **(c,d)** Principal coordinate analysis (PCoA) based on BrayCurtis distance at 3 weeks **(c)** and 3 months **(d)**. Each dot represents one sample; percentages in parentheses indicate variance explained by each principal coordinate (PC). PERMANOVA revealed significant structural divergence between groups at 3 weeks (*R*^2^ = 0.06, *p* = 0.013) and 3 months (*R*^2^ = 0.21, *p* = 0.001). PERMANOVA, permutational multivariate analysis of variance; SDT3w, samples collected 3 weeks postoperatively from the stent-based diversion technique group; SDT3m, samples collected 3 months postoperatively from the stent-based diversion technique group; PDI3w, samples collected 3 weeks postoperatively from the prophylactic double-lumen ileostomy group; PDI3m, samples collected 3 months postoperatively from the prophylactic double-lumen ileostomy group. Color coding: blue (SDTpre), green (SDT3w), purple (SDT3m), orange (PDIpre), red (PDI3w), brown (PDI3m), the color scheme for subsequent image groups matches the above.

Gut microbiota diversity was compared between the two groups preoperatively and at 3 weeks and 3 months postoperatively. Baseline comparison (SDTpre vs. PDIpre) showed no significant differences in α-diversity (Obs, Chao1, Shannon, and Simpson indices) or β-diversity (Bray–Curtis distance at the species level) between groups (all *p* > 0.05; Supplementary Figures S1, S2), indicating comparable preoperative microbial profiles. At 3 weeks postoperatively (SDT3w vs. PDI3w), significant intergroup differences emerged in overall diversity and evenness (Shannon and Simpson indices, *p* < 0.05; Figure 1a) and β-diversity (PERMANOVA, *p* < 0.05; Figure 1c). By 3 months (SDT3m vs. PDI3m), species richness (Obs and Chao1 indices) was significantly lower in the PDI group than in the SDT group (*p* < 0.05; Figure 1b), whereas overall diversity and evenness (Shannon and Simpson indices) did not differ significantly between groups (Supplementary Figure S3). Structural divergence persisted at the β-diversity level (PERMANOVA, *p* < 0.05; Figure 1d).

**Figure 2:**
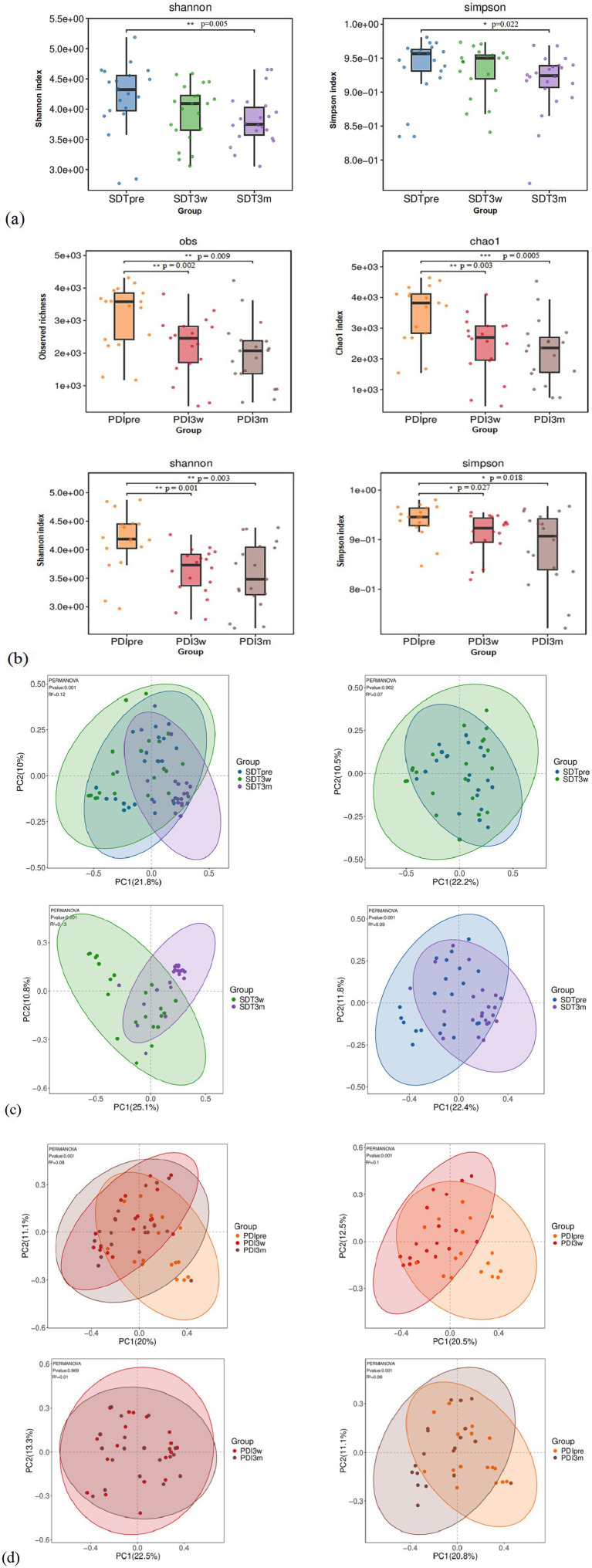
Within-group longitudinal changes in gut microbiota diversity. **(a,b)** α-diversity indices (Shannon, Simpson, Obs, and Chao1) in the SDT **(a)** and PDI **(b)** groups at preoperation (Pre), 3 weeks (3w), and 3 months (3 m) postoperatively. Box plots show the median, interquartile range, and distribution. Statistical significance is indicated above the plots (*p < 0.05, **p < 0.01, ***p < 0.001). **(c,d)** PCoA based on Bray–Curtis distance illustrating structural shifts across the three time points in the SDT **(c)** and PDI **(d)** groups. Each dot represents one sample; percentages in parentheses indicate variance explained by each principal coordinate (PC). Between-time-point differences were assessed by PERMANOVA (999 permutations), with *p*-values and *R*^2^ values shown in the panels. SDTpre, samples collected 1 day preoperatively from the stent-based diversion technique group; SDT3w, samples collected 3 weeks postoperatively from the stent-based diversion technique group; SDT3m, samples collected 3 months postoperatively from the stent-based diversion technique group; PDIpre, samples collected 1 day preoperatively from the prophylactic double-lumen ileostomy group; PDI3w, samples collected 3 weeks postoperatively from the prophylactic double-lumen ileostomy group; PDI3m, samples collected 3 months postoperatively from the prophylactic double-lumen ileostomy group.

Intra-group α-diversity analysis revealed that the SDT group exhibited significantly reduced Shannon and Simpson indices at the species level at 3 months compared with baseline (Figure 2a), whereas other α-diversity metrics (Obs and Chao1) showed no significant change (Supplementary Figure S4). In the PDI group, Obs, Chao1, and Simpson indices were significantly lower than preoperative values at both 3 weeks and 3 months (*p* < 0.05; Figure 2b), indicating a progressive temporal decline in species richness and evenness. β-diversity analysis demonstrated significant structural shifts across all three time points within the SDT group (*p* < 0.05; Figure 2c), with the most pronounced separation observed between 3 months and both baseline (*p* < 0.05) and 3 weeks (*p* < 0.05). In the PDI group, microbial communities at 3 weeks and 3 months both differed significantly from baseline (*p* < 0.05; Figure 2d), whereas no significant structural change occurred between 3 weeks and 3 months (*p* > 0.05; Figure 2d). In addition, within the PDI group, α- and β-diversity did not differ significantly between patients with and without AS at 3 months (*p* > 0.05), precluding further microbial stratification by this clinical endpoint. (Supplementary Figure S10).

### 3.3 Postoperative microbial dynamics and differential species enrichment between SDT and PDI groups

**Figure 3:**
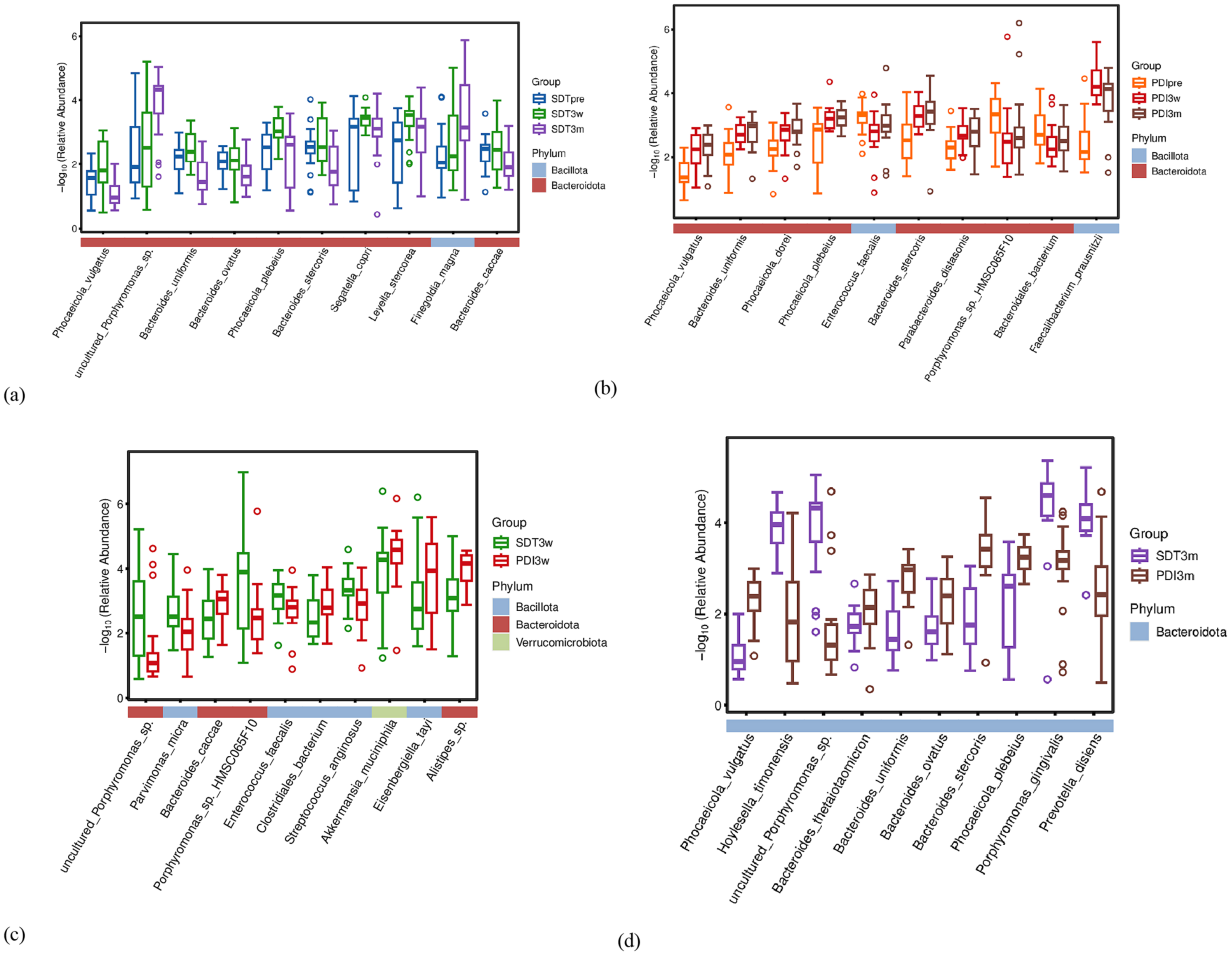
Characteristics of microbial dynamics and differential species abundance between the SDT group and PDI group. Four box plots display log_10_-transformed relative abundances. Boxes represent the interquartile range (IQR), horizontal lines indicate medians, whiskers extend to 1.5× IQR, and points beyond are outliers. The *y*-axis shows log_10_-transformed relative abundance; colored bars below the *x*-axis indicate phylum-level classification. Color coding: blue (SDTpre), green (SDT3w), purple (SDT3m), orange (PDIpre), red (PDI3w), and brown (PDI3m), consistent with other figures. **(a,b)** Intra-group longitudinal comparison: the top 10 differentially abundant species (*p* < 0.05, Kruskal–Wallis for three time points or Wilcoxon signed-rank test for two time points) are shown in descending order of mean abundance. **(a)** SDT group. **(b)** PDI group. **(c,d)** Between-group comparison: the top 10 differentially abundant species (*p* < 0.05, Wilcoxon rank-sum test) are shown in descending order of mean abundance. **(c)** SDT3w versus PDI3w. **(d)** SDT3m versus PDI3m.

Based on the observed microbial diversity differences, we investigated the dynamic trajectories of postoperative gut microbiota and identified differentially abundant species using the Wilcoxon rank-sum test (between-group) and Kruskal–Wallis test (intra-group three time points), with *p* < 0.05 as the significance threshold. The top 10 differentially abundant species at the species level were visualized as boxplots (Figure 3), with individual species plots provided in Supplementary Figures S5–S8.

In the SDT group, *Leyella stercorea* and *Finegoldia magna* (*Firmicutes*) exhibited significant decreases at 3 months postoperatively. Several Bacteroidota species showed time-dependent dynamics: *Phocaeicola vulgatus*, *Bacteroides uniformis*, and *Phocaeicola plebeius* decreased at 3 weeks but recovered by 3 months, whereas *Bacteroides ovatus*, *Bacteroides caccae*, and *Bacteroides stercoris* progressively increased over time. These species are predominantly beneficial gut commensals. Conversely, *uncultured_Porphyromonas_sp*., a conditional pathogen, was abundant preoperatively and at 3 weeks but significantly declined by 3 months (Figure 3a).

In the PDI group, intra-group shifts were also dominated by *Firmicutes* and *Bacteroidota*. *Enterococcus faecalis* sharply increased at 3 weeks and subsequently declined, whereas *Faecalibacterium prausnitzii* markedly decreased at 3 weeks. Several *Bacteroidota* species, including *Bacteroides uniformis*, *Bacteroides dorei*, *Parabacteroides distasonis*, and *Phocaeicola vulgatus*, showed progressive declines over the postoperative period (Figure 3b).

Cross-sectional between-group comparison showed that the top 10 differential species comprised four *Bacteroidota* and five *Firmicutes* members at 3 weeks postoperatively. Within *Bacteroidota*, *Alistipes* sp. and *Bacteroides caccae* were enriched in the SDT group, whereas *uncultured_Porphyromonas_sp.* and *Porphyromonas sp. HMSC065F10* were enriched in the PDI group. Within *Firmicutes*, the PDI group was dominated by *Parvimonas micra* and *Streptococcus anginosus*, whereas the SDT group showed enrichment of *Clostridiales bacterium* and the rare species *Eisenbergiella tayi*. Notably, *Akkermansia muciniphila* (*Verrucomicrobiota*) was significantly more abundant in the SDT group (Figure 3c). These findings indicate that the two diversion strategies induced divergent microbial profiles as early as 3 weeks postoperatively.

By 3 months, six *Bacteroidota* species were significantly more abundant in the SDT group, including *Phocaeicola vulgatus*, *Bacteroides thetaiotaomicron*, *Bacteroides stercoris*, *Bacteroides uniformis*, *Bacteroides ovatus*, and *Phocaeicola plebeius*. In contrast, the PDI group showed enrichment of multiple *Prevotellaceae* members, including *Hoylesella timonensis*, *Porphyromonas gingivalis*, *uncultured_Porphyromonas_sp.*, and *Prevotella disiens* (Figure 3d).

**Figure 4:**
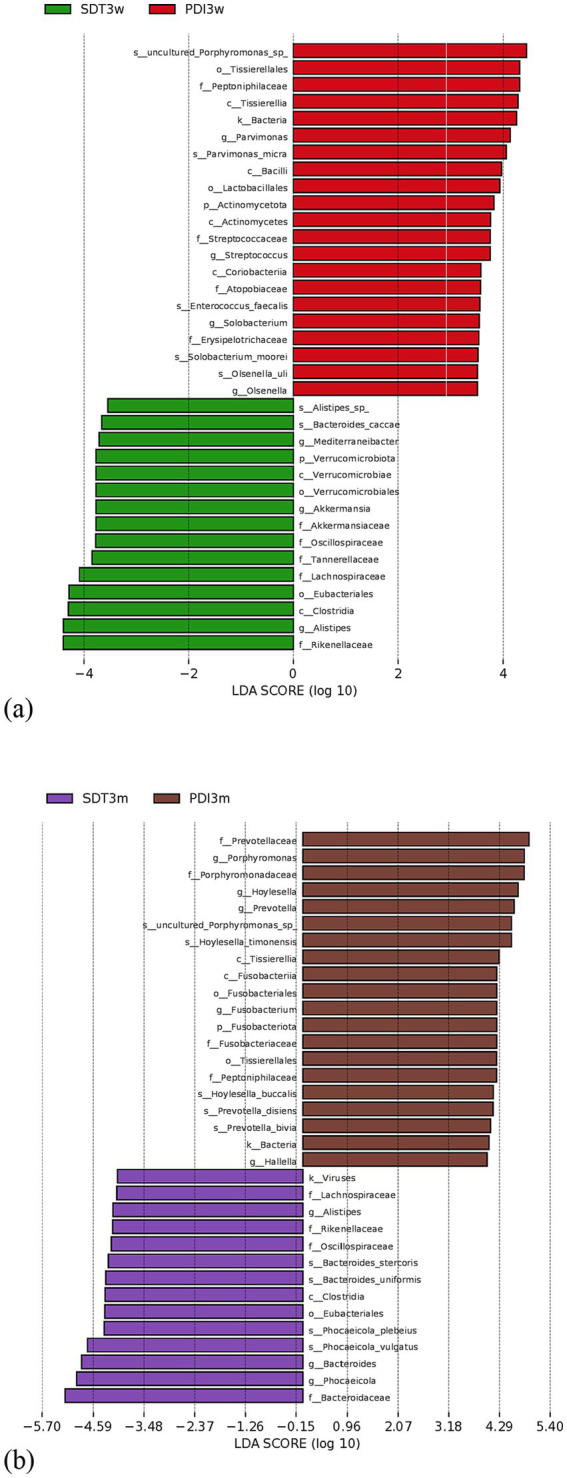
Differential microbial biomarkers identified by LEfSe analysis at 3 weeks and 3 months postoperatively. Bar plots show significantly discriminative taxa (LDA score > 3.5, *p* < 0.05). **(a)** Taxa enriched in the PDI group (red) and SDT group (green) at 3 weeks. **(b)** Taxa enriched in the PDI group (brown) and SDT group (purple) at 3 months. Bar length represents the LDA score (log10-transformed linear discriminant analysis effect size), with longer bars indicating a greater effect size for discriminating between groups. Taxonomic prefixes: p, phylum; c, class; o, order; f, family; g, genus; s, species.

To precisely identify discriminative taxa, we performed species-level LEfSe analysis (LDA > 3.0, *p* < 0.05). At 3 weeks, 36 differential taxa were identified (Figure 4a, Supplementary Figure S9a, Supplementary Table 1). The SDT-enriched taxa primarily included *Akkermansia*, *Alistipes*, *Oscillospiraceae*, *Bacteroides caccae*, *Lachnospiraceae*, and *Rikenellaceae*, whereas the PDI group was characterized by *Parvimonas micra*, *Enterococcus faecalis*, *Streptococcus*, *Peptoniphilaceae*, and *Porphyromonas*.

At 3 months, 33 taxonomic clades showed significant between-group differences (LDA > 3.5, *p* < 0.05), with 13 enriched in the SDT group and 20 in the PDI group (Figure 4b, Supplementary Figure S9b, Supplementary Table 2). The SDT group was predominantly enriched in *Bacteroidaceae*, *Bacteroides*, *Phocaeicola* (including *P. vulgatus* and *P. plebeius*), as well as potential butyrate producers including *Lachnospiraceae*, *Oscillospiraceae*, and *Alistipes* spp. The PDI group showed enrichment of *Prevotellaceae*, *Porphyromonas*, *Hoylesella* (including *H. timonensis* and *H. buccalis*), and notably, *Fusobacteriota* and its member *Fusobacterium*. Additionally, genitourinary-associated *Prevotella bivia* and *Prevotella disiens* were specifically enriched in the PDI group. These findings demonstrate markedly divergent microbial compositions at 3 months postoperatively; however, their clinical implications require further validation through functional analysis and long-term follow-up.

### 3.4 Differential KEGG functional pathways between SDT and PDI groups

**Figure 5:**
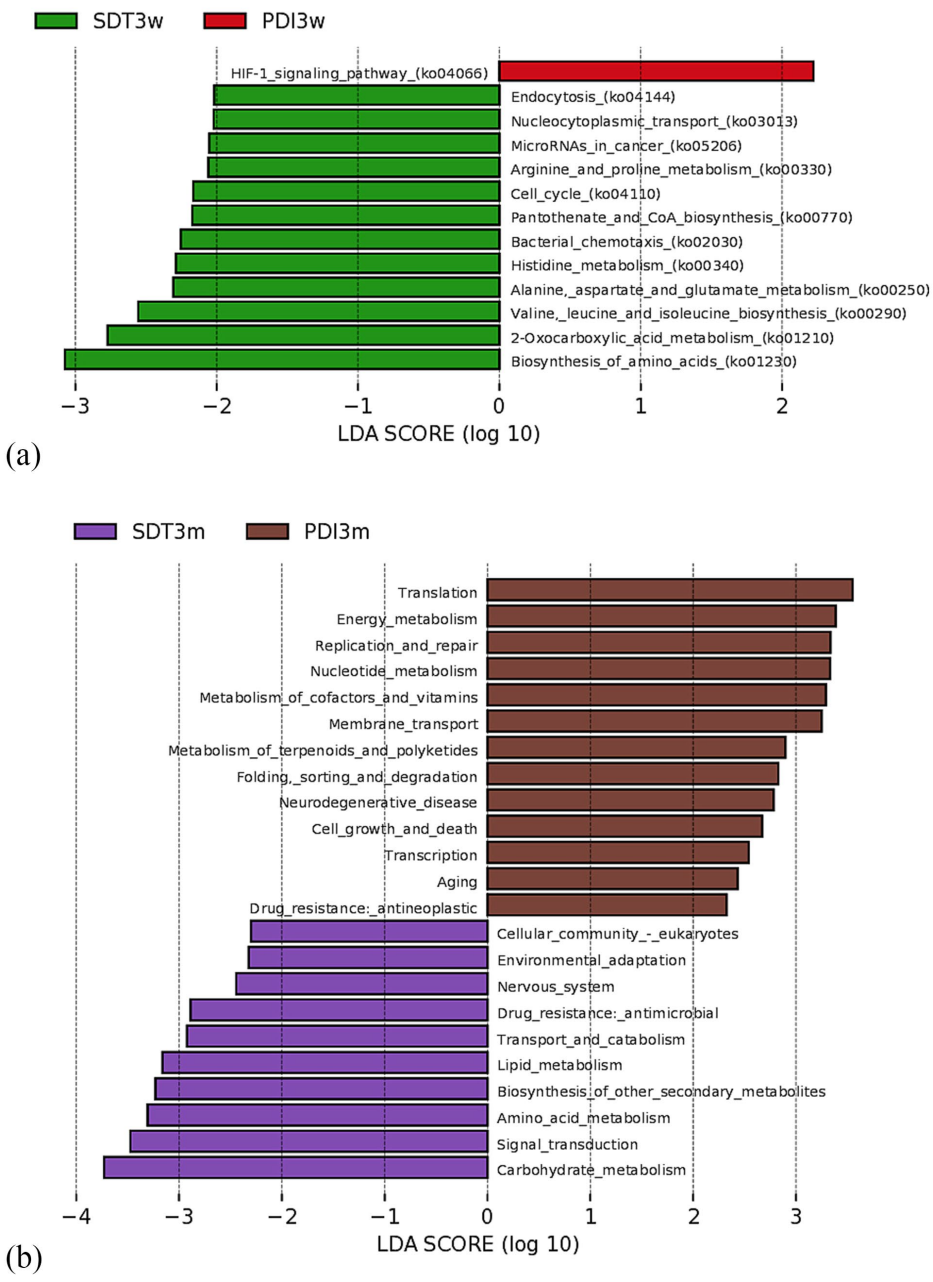
Differential KEGG pathways identified by LEfSe analysis between the SDT and PDI groups. **(a)** Level 3 KEGG pathways at 3 weeks postoperatively (SDT3w vs. PDI3w, LDA score > 2.0, *p* < 0.05). Green bars: pathways enriched in the SDT group; red bars: pathways enriched in the PDI group. **(b)** Level 2 KEGG pathways at 3 months postoperatively (SDT3m vs. PDI3m, LDA score > 2.0, *p* < 0.05). Purple bars: pathways enriched in the SDT group; brown bars: pathways enriched in the PDI group. Bar length represents the LDA score, with longer bars indicating a larger effect size for discriminating between groups.

LEfSe analysis of KEGG pathways was performed to identify functional differences between the two groups. At 3 weeks postoperatively, analysis at KEGG level 3 revealed 12 significantly differential pathways (LDA > 2.0, *p* < 0.05; Figure 5a, Supplementary Table 4). Eleven pathways were enriched in the SDT group, predominantly involving amino acid metabolism and fundamental biosynthetic processes, with amino acid biosynthesis showing the highest enrichment, followed by 2-oxocarboxylic acid metabolism, branched-chain amino acid biosynthesis, and alanine/aspartate/glutamate metabolism. Bacterial chemotaxis and cell cycle pathways were also enriched in the SDT group. In contrast, only one pathway—HIF-1 signaling—was significantly enriched in the PDI group.

At 3 months, no significant differences were detected at KEGG level 3. Subsequent LEfSe analysis at KEGG level 2 identified 23 differential pathways (LDA > 2.0, *p* < 0.05), with 10 enriched in the SDT group and 13 in the PDI group (Figure 5b, Supplementary Table 4). SDT-enriched pathways were predominantly metabolism-related, encompassing carbohydrate metabolism, signal transduction, amino acid metabolism, and lipid metabolism, alongside antimicrobial resistance. PDI-enriched pathways were primarily fundamental life-sustaining processes, including translation, energy metabolism, replication and repair, and nucleotide metabolism. Notably, host disease-related pathways, such as neurodegenerative diseases and aging, were also enriched in the PDI group.

Compared with the 12 differential KEGG level 3 pathways observed at 3 weeks, the functional divergence at 3 months shifted from specific pathways to broader functional categories, suggesting a progressive temporal remodeling of the gut microbiota during postoperative recovery.

## 4 Discussion

LAR is the standard procedure for mid-to-low rectal cancer; however, AL, occurring in 1.5–20% of cases (Degiuli et al., 2022; Bekki et al., 2023; Rutegård et al., 2024), remains one of the most severe complications. AL is classified into three grades (A, B, and C), with grade C being the most critical, potentially leading to intra-abdominal or pelvic infection, sepsis, and even death, necessitating surgical intervention (Rahbari et al., 2010). To mitigate AL risk, PDI is widely employed to effectively divert intestinal contents (Palumbo et al., 2019; Albulescu et al., 2023). However, PDI is associated with considerable clinical drawbacks: it requires a secondary reversal procedure, imposing additional patient burden; stoma-related complications (prolapse, parastomal hernia, obstruction, bleeding, etc.) occur frequently; and prolonged fecal diversion may cause disuse atrophy of the intestinal tract, increasing the risk of AS (Gilshtein et al., 2022; Jin and Yong, 2022). Therefore, how to effectively prevent AL while avoiding stoma-related morbidities remains an urgent clinical challenge in colorectal surgery.

To circumvent these limitations, our team participated in the development of the SDT This device effectively diverts intestinal contents during the high-risk period of 3–4 weeks postoperatively before spontaneous degradation, thereby protecting the anastomosis while eliminating the need for a secondary procedure. Previous studies have demonstrated the biosafety of SDT and its potential as an alternative to PDI(Song et al., 2022; Tong et al., 2024); further technical refinement combining a biodegradable stent with a protective film sleeve significantly reduced the overall complication rate compared with PDI (18.9% vs. 51.4%, *p* < 0.05) (Chen et al., 2024). Nevertheless, the differential impact of these two diversion strategies on postoperative gut microbiota remains poorly characterized. This study aimed to compare the effects of SDT and PDI on postoperative gut microbiota and clinical outcomes in rectal cancer patients. By analyzing the dynamic trajectories of microbial composition and functional pathways, we sought to explore the potential advantages of SDT in promoting postoperative intestinal recovery, offering novel insights for optimizing perioperative management strategies.

Clinical outcomes analysis revealed longer operative time in the SDT group than in the PDI group (245 min vs. 204 min, *p* < 0.05). However, this difference is unlikely to explain the observed microbial divergence, as preoperative microbiota profiles were comparable between groups and the differential ecological trajectories emerged postoperatively, temporally aligning with the diversion period rather than intraoperative stress. Total hospitalization costs were significantly higher in the PDI group (8.00 vs. 5.10 ten-thousand CNY, *p* < 0.05) due to stoma-related complications and reversal surgery. AL rates were similarly low (one case each, *p* > 0.05), whereas AS occurred significantly less frequently with SDT (4.76% vs. 31.58%, *p* < 0.05). AS, a serious complication of ileostomy with an incidence of 2.5–30% (Biraima et al., 2016; Sartori et al., 2019; Lin et al., 2022; Cong et al., 2023), is classified by severity into Grades I–III (Llorach-Perucho et al., 2024) and by morphology into membranous, tubular, and diffuse subtypes. Membranous stricture occurs in 20–30% of patients after low anterior resection with PDI and results from prolonged absence of fecal transit (Placer et al., 2010). Diagnostic criteria include inability to pass the index finger or a 12-mm colonoscope through the anastomosis, or difficult passage accompanied by obstructive symptoms (Fu and Hao, 2014; Xiyu et al., 2018; Yuliuming Wang, 2020; Tang, 2021; Jinjin et al., 2024). In this study, 7 patients met these criteria: 1 in the SDT group (Grade II) and 6 in the PDI group (all membranous). This distribution is consistent with the established pathophysiology of diversion-associated membranous stricture rather than microbial factors (Placer et al., 2010). Collectively, these findings indicate superior postoperative outcomes with SDT.

Metagenomic sequencing revealed that surgical significantly reduced α-diversity in both groups, consistent with previous reports (Deng et al., 2018). However, divergent recovery trajectories suggest that fecal diversion modality may influence postoperative microbiota succession.

Three weeks postoperatively marked a critical divergence between the two diversion strategies. The PDI group exhibited significant enrichment of potential opportunistic pathogens, including *Enterococcus faecalis*, *Parvimonas micra*, and *Streptococcus. E. faecalis* can cause intra-abdominal infections and bacteremia under specific conditions (Fisher and Phillips, 2009), whereas *P. micra* has been implicated in colorectal cancer progression (Tsoi et al., 2017). This pathogen enrichment likely reflects alterations in the distal intestinal microenvironment induced by complete fecal diversion: absent intestinal contents reduce nutrient availability while elevated intraluminal oxygen tension favors facultative anaerobe expansion (Baek et al., 2014). Conversely, the SDT group maintained partial intestinal content transit, preserving a more physiologically compatible distal microenvironment and enabling enrichment of beneficial taxa such as *Akkermansia*, *Lachnospiraceae*, and *Alistipes* (Moschen et al., 2016; Cani et al., 2022; Zhang et al., 2023). *Akkermansia* enhances barrier function through mucin degradation and short-chain fatty acid production (Derrien et al., 2017); *Lachnospiraceae*, as principal butyrate producers, supply energy to intestinal epithelial cells and exert anti-inflammatory and reparative effects (Vital et al., 2017); and *Alistipes* is similarly associated with anti-inflammatory and metabolic benefits (Moschen et al., 2016). Both procedures entail comparable anatomical resection (LAR); the fundamental distinction lies in diversion modality. PDI necessitates a secondary reversal procedure, whereas the SDT stent spontaneously degrades within 3–4 weeks, obviating additional surgery. This differential intervention may reshape the intestinal microenvironment and consequently influence microbiota composition and recovery trajectories.

Functionally, the SDT group exhibited enrichment of amino acid metabolism and bacterial chemotaxis pathways at 3 weeks, indicative of active anabolic states. The PDI group showed higher abundance of HIF-1 signaling pathway-related genes. HIF-1 is a core pathway through which host cells respond to hypoxic environments (Di Mattia et al., 2024), and its expression level in anastomotic tissue is closely associated with postoperative healing quality. Reduced HIF-1α expression may alleviate local inflammation and improve tissue repair (Strowitzki et al., 2021; Wang et al., 2024). Ischemia and hypoxia can activate HIF-1α, enhance profibrotic pathways, and ultimately lead to AS (Yavuz et al., 2025). The detection of HIF-1 pathway genes in metagenomic data may originate from residual host epithelial cell DNA or host pathway activation induced by localized intestinal hypoxia, indirectly suggesting that the PDI group may experience localized intestinal hypoxia during the early postoperative period. However, the direct association between this finding and microbiota function remains unclear.

By 3 months, intergroup differences became more pronounced. In the SDT group, *Finegoldia magna*—a virulent opportunistic pathogen (Boyanova et al., 2016)—significantly decreased, whereas six beneficial Bacteroidota species with anti-inflammatory and mucosal repair properties (e.g., *Phocaeicola vulgatus*, *Bacteroides uniformis*) increased (Wexler, 2007). The oral-derived conditional pathogen *uncultured_Porphyromonas_sp.* (Kerdreux et al., 2023; Dillard et al., 2025) exhibited only transient elevation at 3 weeks before declining by 3 months, suggesting that SDT may create a competitive advantage for beneficial colonization.

The PDI group displayed distinct characteristics at 3 months. Core functional taxa such as *Parabacteroides distasonis* (Zhao et al., 2023) and the key butyrate-producer *Faecalibacterium prausnitzii* decreased, potentially reducing short-chain fatty acid production and compromising intestinal barrier function (Miquel et al., 2013). Previous studies have demonstrated that fecal butyrate levels are significantly lower in patients with anastomotic leakage, highlighting the critical role of SCFAs in anastomotic healing (Boatman et al., 2024). Concurrently, the PDI group showed enrichment of oral/genitourinary-associated bacteria, including Prevotellaceae, *Porphyromonas* (Kerdreux et al., 2023), and *Hoylesella*. These taxa are frequently regarded as markers of impaired barrier function or ecological collapse and have been linked to inflammatory states in inflammatory bowel disease and colorectal cancer (Chen et al., 2022; Chen et al., 2025). They likely represent selective expansion of low-abundance commensals driven by anatomical alterations and microenvironmental changes (e.g., oxygen concentration, pH, substrate availability) following diversion (Losno et al., 2021; Catalan et al., 2024; Xu et al., 2025), rather than true ectopic colonization. Notably, *Fusobacterium* was specifically enriched in the PDI group and has been implicated in colorectal cancer progression through FadA-mediated activation of β-catenin signaling (Rubinstein et al., 2013).

Functionally, differences shifted from specific pathways (Level 3) to broader functional categories (Level 2) by 3 months. The SDT group was enriched in carbohydrate, amino acid, and lipid metabolism pathways, consistent with its taxonomic composition (Kanehisa et al., 2023), whereas the PDI group showed enrichment of fundamental life-sustaining pathways including translation, energy metabolism, and replication/repair. These findings suggest that SDT may guide the microbiota toward a more resilient, health-associated ecological state.

This study carries important clinical implications. The enrichment of butyrate-producing Lachnospiraceae in the SDT group may support intestinal barrier integrity (Parada Venegas et al., 2019). The enrichment of opportunistic pathogens and *Fusobacterium* in the PDI group warrants closer follow-up or targeted microecological intervention, particularly given the potential impact of *Fusobacterium* on long-term oncological outcomes (Kostic et al., 2013; Rubinstein et al., 2013; Zhao et al., 2022; Lu et al., 2024). The association between intestinal translocation of oral microbiota and colorectal cancer risk provides additional perspective on the long-term implications for PDI patients (Mo et al., 2022).

In conclusion, differences in fecal diversion modality and the duration until restoration of transanal defecation may reshape the intestinal microenvironment and consequently alter microbiota composition. However, the direct causal relationship between these microbial differences and clinical outcomes remains to be established.

This study has several limitations. First, the relatively small sample size (*n* = 40) and single-center design may introduce selection bias. Second, the follow-up duration of 12 months precludes direct association of microbiota changes with long-term hard endpoints. Third, potential confounders such as diet were not fully controlled. Fourth, functional analyses were based on KEGG database annotations and remain predictive, awaiting validation by metabolomics or other direct evidence. Future studies should validate these findings in larger multicenter cohorts with extended follow-up, integrate multi-omics approaches to elucidate underlying molecular mechanisms, explore the therapeutic potential of microecological interventions such as probiotics or fecal microbiota transplantation in PDI patients, and determine whether specific microbial signatures may serve as early biomarkers for postoperative complications, including AS.

## 5 Conclusion

This study demonstrates that, compared with prophylactic PDI, SDT significantly reduces AS incidence (4.76% vs. 31.58%, *p* < 0.05) and hospitalization costs. Metagenomic analysis revealed that SDT better preserves postoperative gut microbiota diversity and stability, with recovery toward baseline by 3 months, whereas PDI exhibits progressive ecological deterioration. SDT enriches beneficial commensals and metabolic pathways involved in amino acid and carbohydrate metabolism, while PDI favors opportunistic pathogens and fundamental life-sustaining functions. These findings support SDT as a clinically effective and microecologically favorable alternative for fecal diversion in rectal cancer surgery.

## Data Availability

The raw metagenomic sequencing data reported in this paper have been deposited in the NCBI Sequence Read Archive (SRA) database under BioProject accession number PRJNA1475036.
