# Supplementary material for: Comparison of the effects of stent-based diversion technique versus prophylactic double-lumen ileostomy on intestinal flora in postoperative patients with rectal cancer
Source: Front Microbiol. 2026 Jul 21;17:1791364. doi: 10.3389/fmicb.2026.1791364 (PMC13436953; doi:10.3389/fmicb.2026.1791364)
Supplement: Supplementary file 1 [file Data_Sheet_1.PDF]

Supplementary Figure

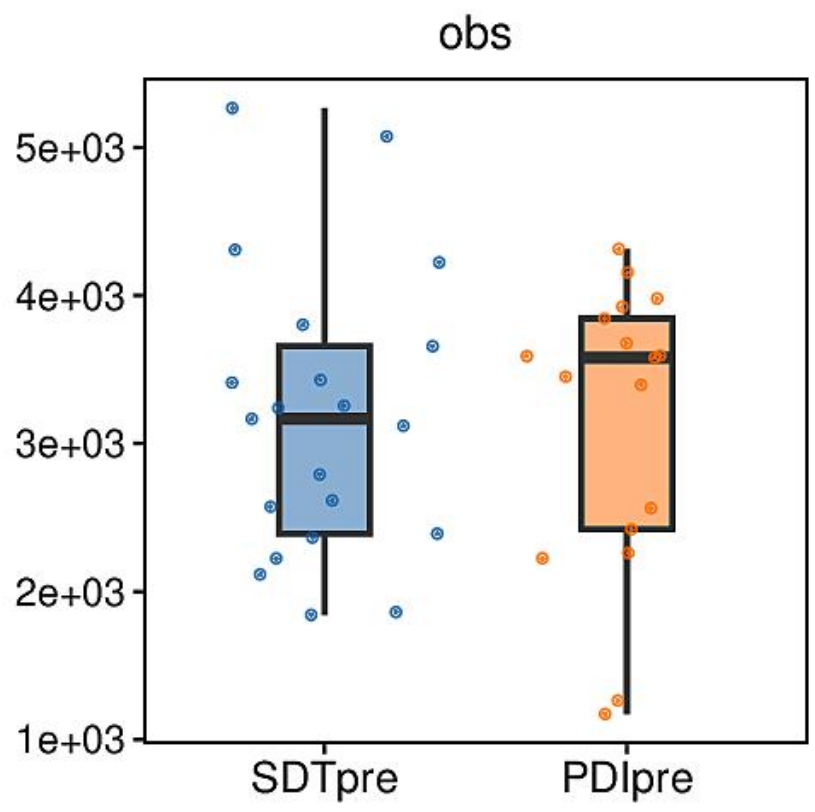

(a)

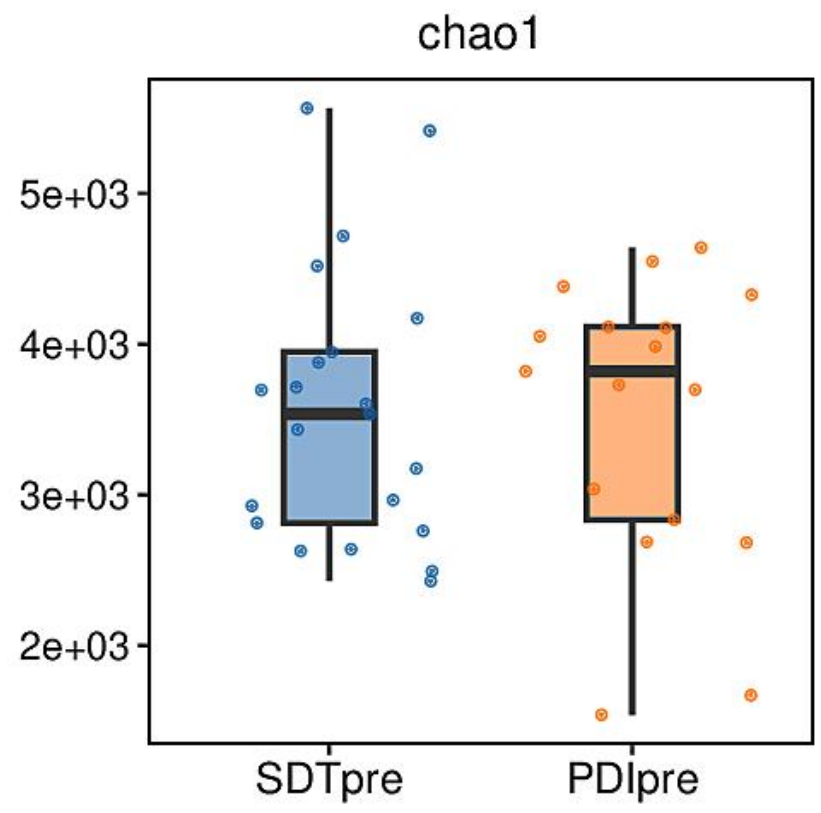

(b)

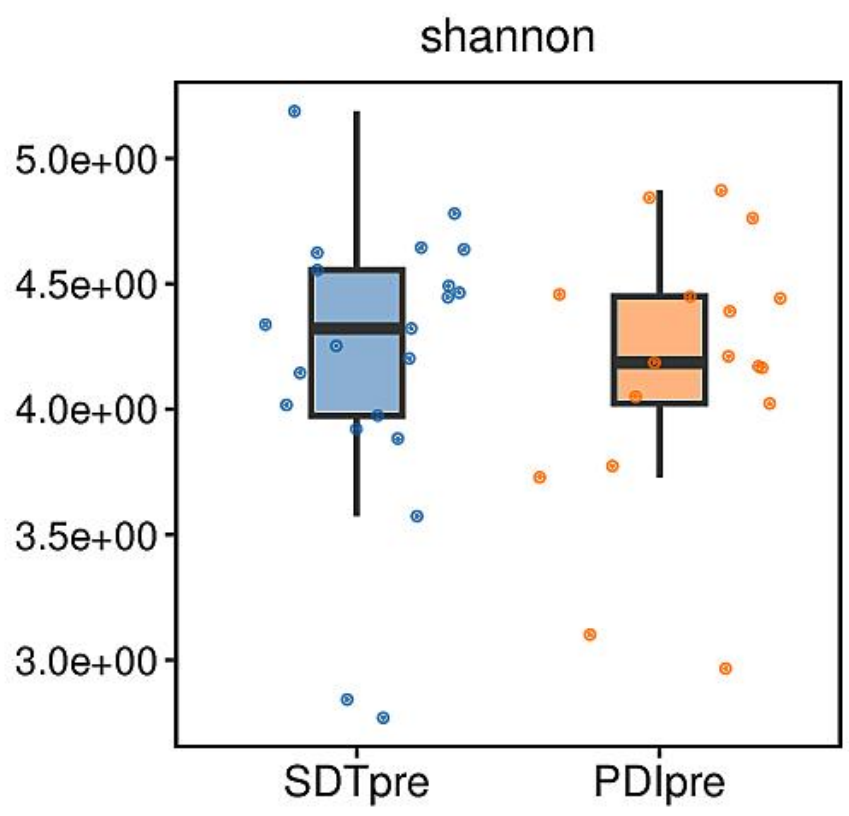

(c)

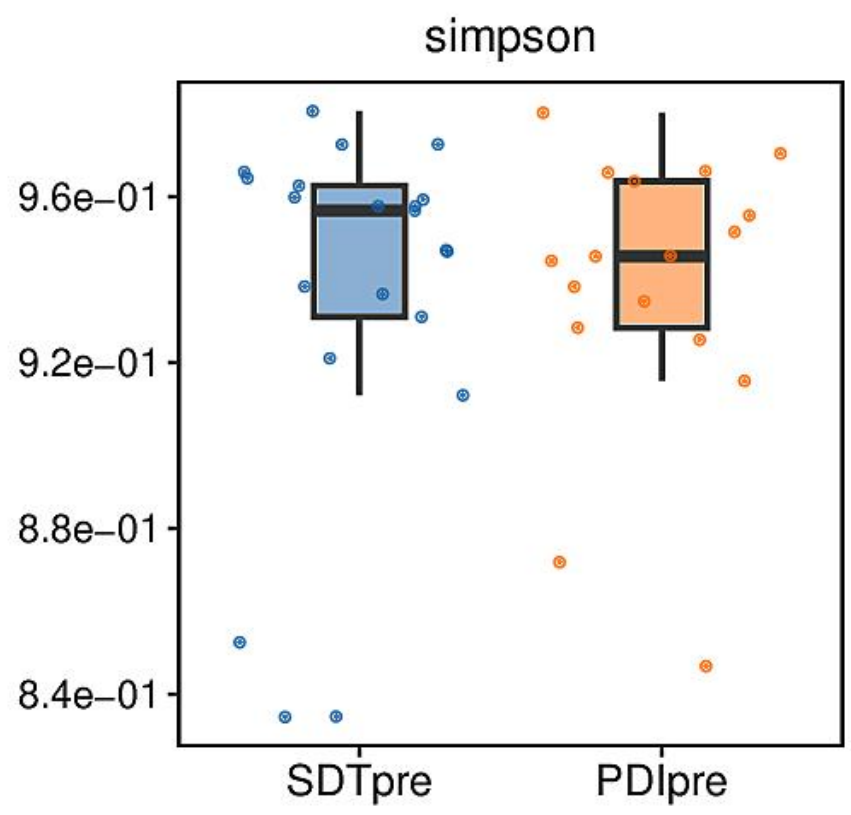

(d)

Figure S1. Comparison of gut microbiota α-diversity between the SDT and PDI groups. (a-d) α-diversity indices (Simpson, Shannon, Obs, and Chao1) at 1 day preoperatively. Box plots show the median, interquartile range, and data distribution. The vertical axis indicates the relative abundance values of species. Significance levels are indicated above the comparisons (* $p < 0.05$, ** $p < 0.01$, *** $p < 0.001$). Abbreviations: SDTpre: Samples collected 1day preoperatively from the stent-based diversion technique group. None of the above showed statistical differences, no significance was indicated. Color coding: blue (SDTpre), green (SDT3w), purple (SDT3m), orange (PDIpre), red (PDI3w), brown (PDI3m), the color scheme for subsequent image groups matches the above.

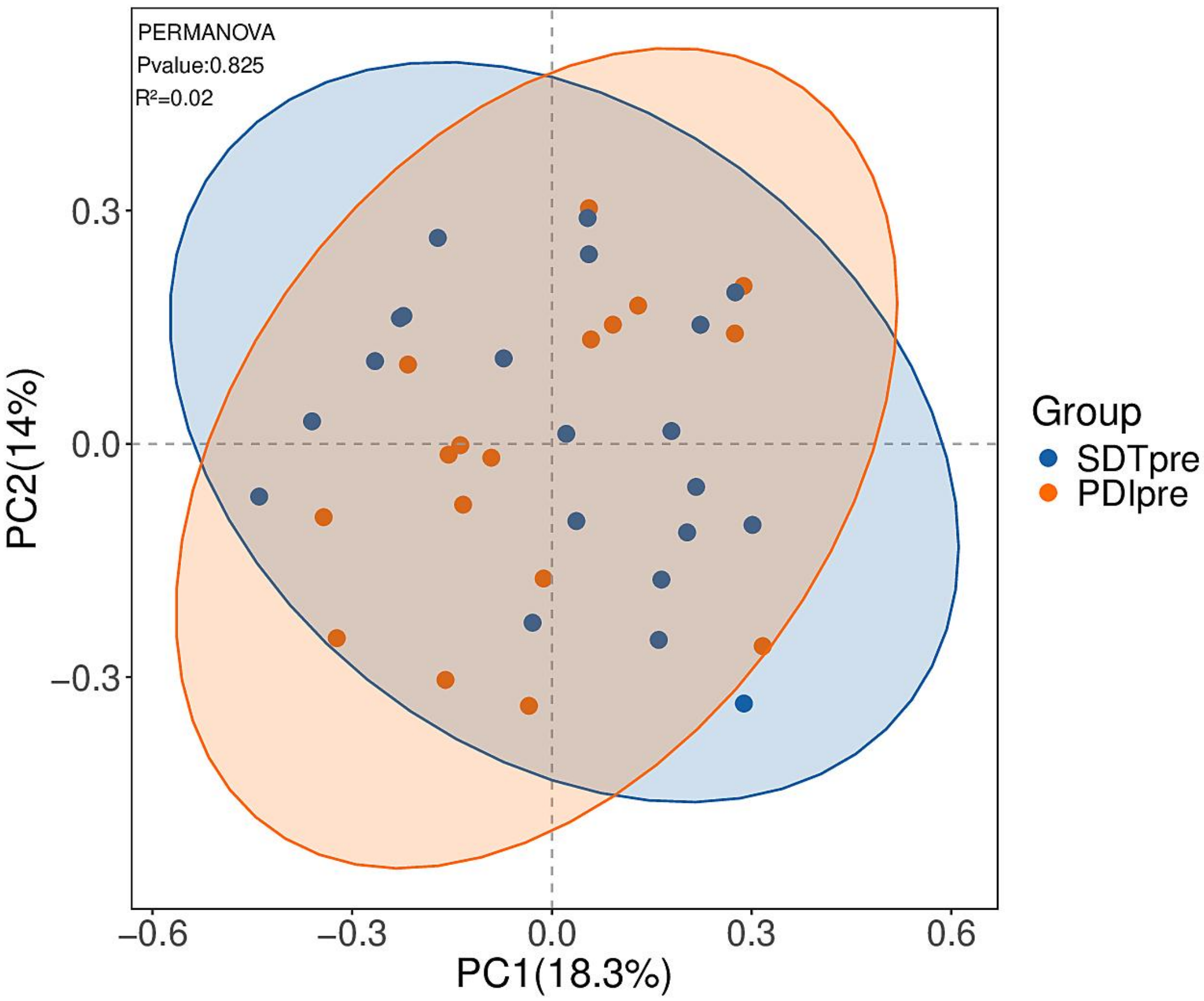

Figure S2. Comparison of gut microbiota β-diversity between the SDT and PDI groups at 1 day preoperatively. Principal coordinate analysis (PCoA) based on Bray - Curtis distance at 1 day preoperatively. Each dot represents one sample; percentages in parentheses indicate variance explained by each principal coordinate (PC). PERMANOVA revealed significant structural divergence between groups at 1 day preoperatively (R^2 = 0.02, p = 0.825). Abbreviations: PERMANOVA = Permutational multivariate analysis of variance; SDTpre: Samples collected 1day preoperatively from the stent-based diversion technique group.

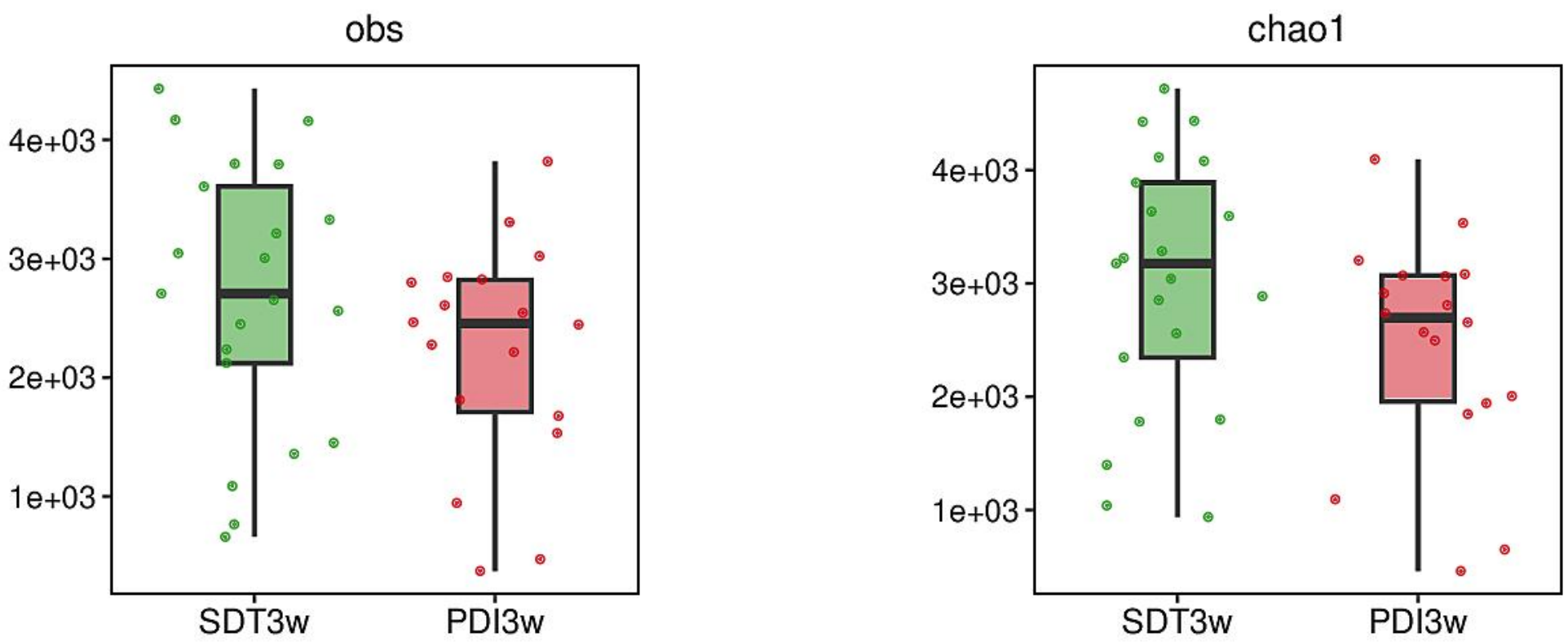

(a) (b)

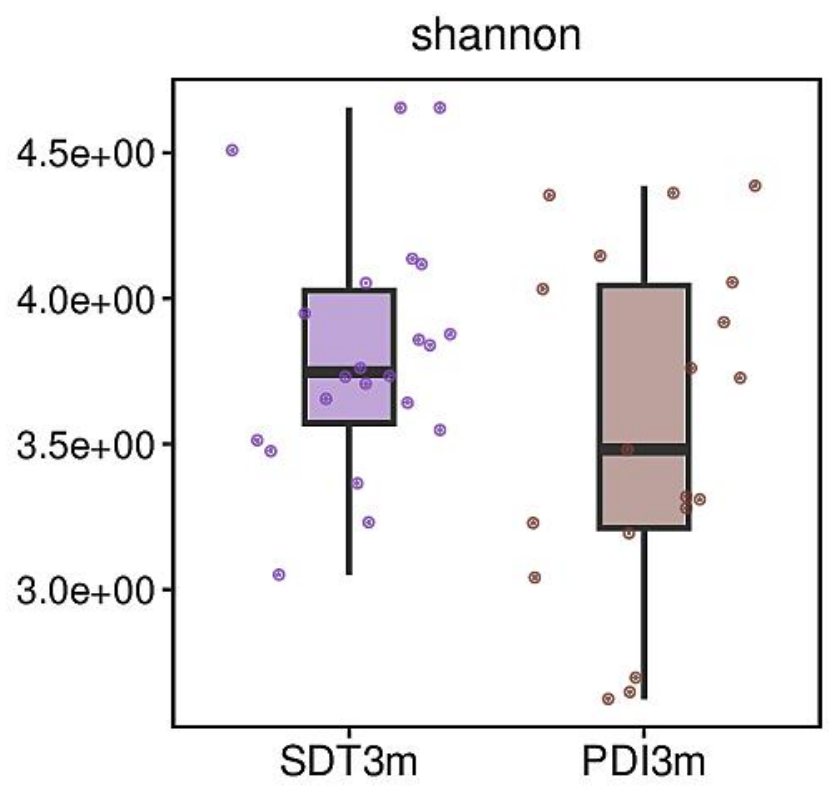

(c)

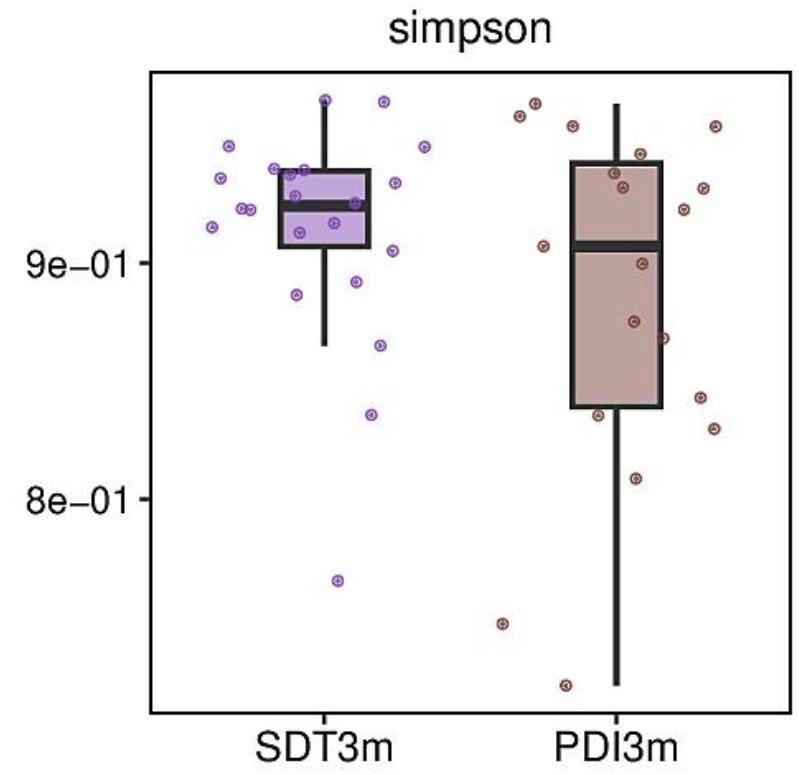

(d)

Figure S3. Comparison of gut microbiota α-diversity between the SDT and PDI groups. (a-b) α-diversity indices (Obs, and Chao1) at 3 weeks postoperatively. (c-d) α-diversity indices (Shannon, and Simpson) at 3 months postoperatively. Box plots show the median, interquartile range, and data distribution. The vertical axis indicates the relative abundance values of species. Significance levels are indicated above the comparisons (*p < 0.05, **p < 0.01, ***p < 0.001). None of the above showed statistical differences, no significance was indicated. Abbreviations: SDT3w: Samples collected 3 weeks postoperatively from the stent-based diversion technique group; SDT3m: Samples collected 3 months postoperatively from the stent-based diversion technique group; PDI3w: Samples collected 3 weeks postoperatively from the prophylactic double-lumen ileostomy group; PDI3m: Samples collected 3 months postoperatively from the prophylactic double-lumen ileostomy group.

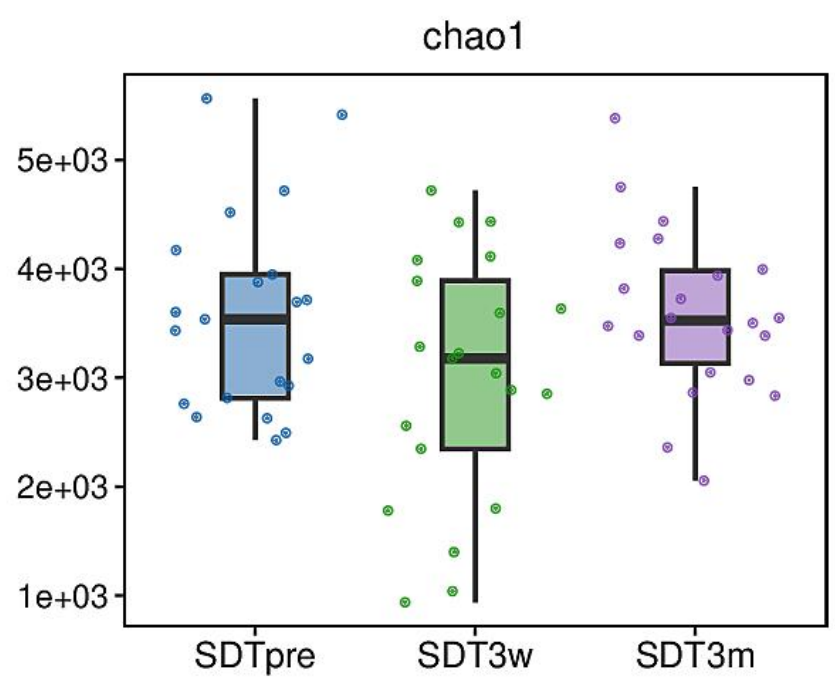

(a)

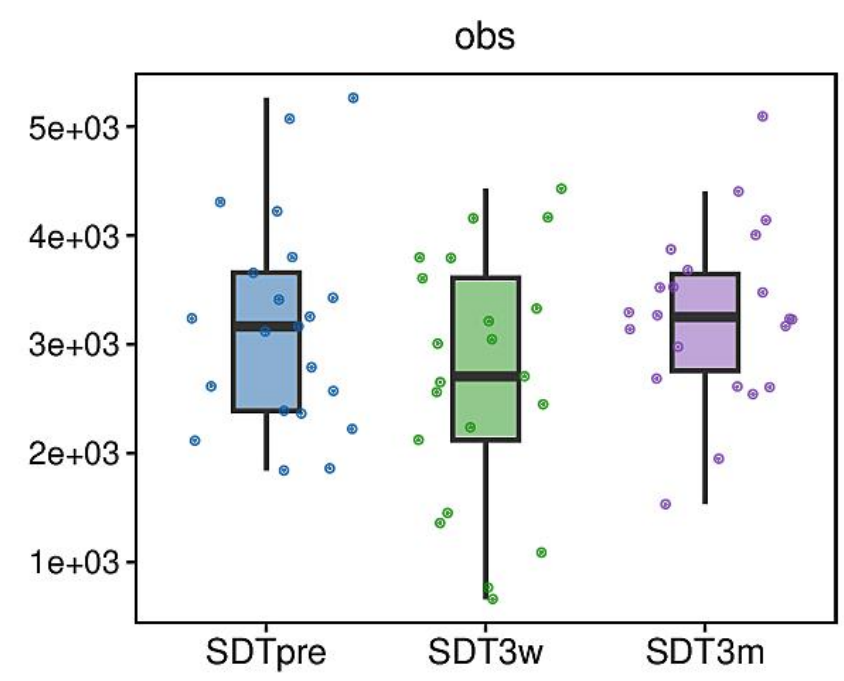

(b)

Figure S4. Comparison of gut microbiota α-diversity within the SDT group at Preoperatively, 3 weeks postoperatively and 3 months postoperatively. Box plots show the median, interquartile range, and data distribution. The vertical axis indicates the relative abundance values of species. Significance levels are indicated above the comparisons (*p < 0.05, **p < 0.01, ***p < 0.001). None of the above showed statistical differences, no significance was indicated. Abbreviations: SDTpre: Samples collected 1day preoperatively from the stent-based diversion technique group. SDT3w: Samples collected 3 weeks postoperatively from the stent-based diversion technique group; SDT3m: Samples collected 3 months postoperatively from the stent-based

diversion technique group; PDIpre: Samples collected 1day preoperatively from the prophylactic double-lumen ileostomy group. PDI3w: Samples collected 3 weeks postoperatively from the prophylactic double-lumen ileostomy group; PDI3m: Samples collected 3 months postoperatively from the prophylactic double-lumen ileostomy group.

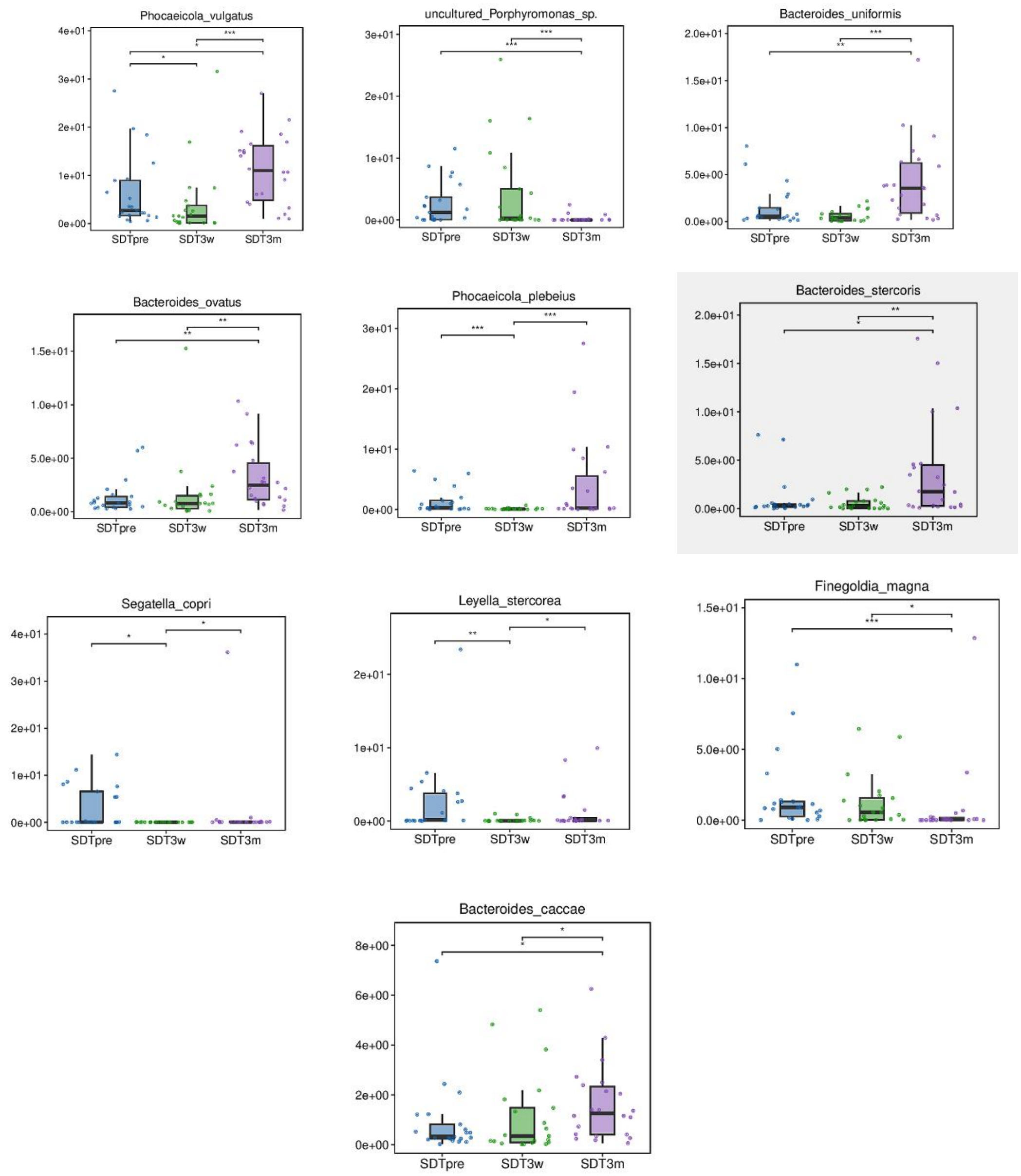

Figure S4. The top ten species with the highest abundance differences within the SDT group at preoperative, 3 weeks postoperatively, and 3 months postoperatively. Box plots show the median, interquartile range, and distribution. Statistical significance is indicated above the plots (*p < 0.05, **p < 0.01, ***p < 0.001). The vertical axis indicates the relative abundance values of species. Abbreviations: SDTpre: Samples collected 1day preoperatively from the stent-based diversion technique group. SDT3w: Samples collected 3 weeks postoperatively from the stent-based diversion technique

group; SDT3m: Samples collected 3 months postoperatively from the stent-based diversion technique group.

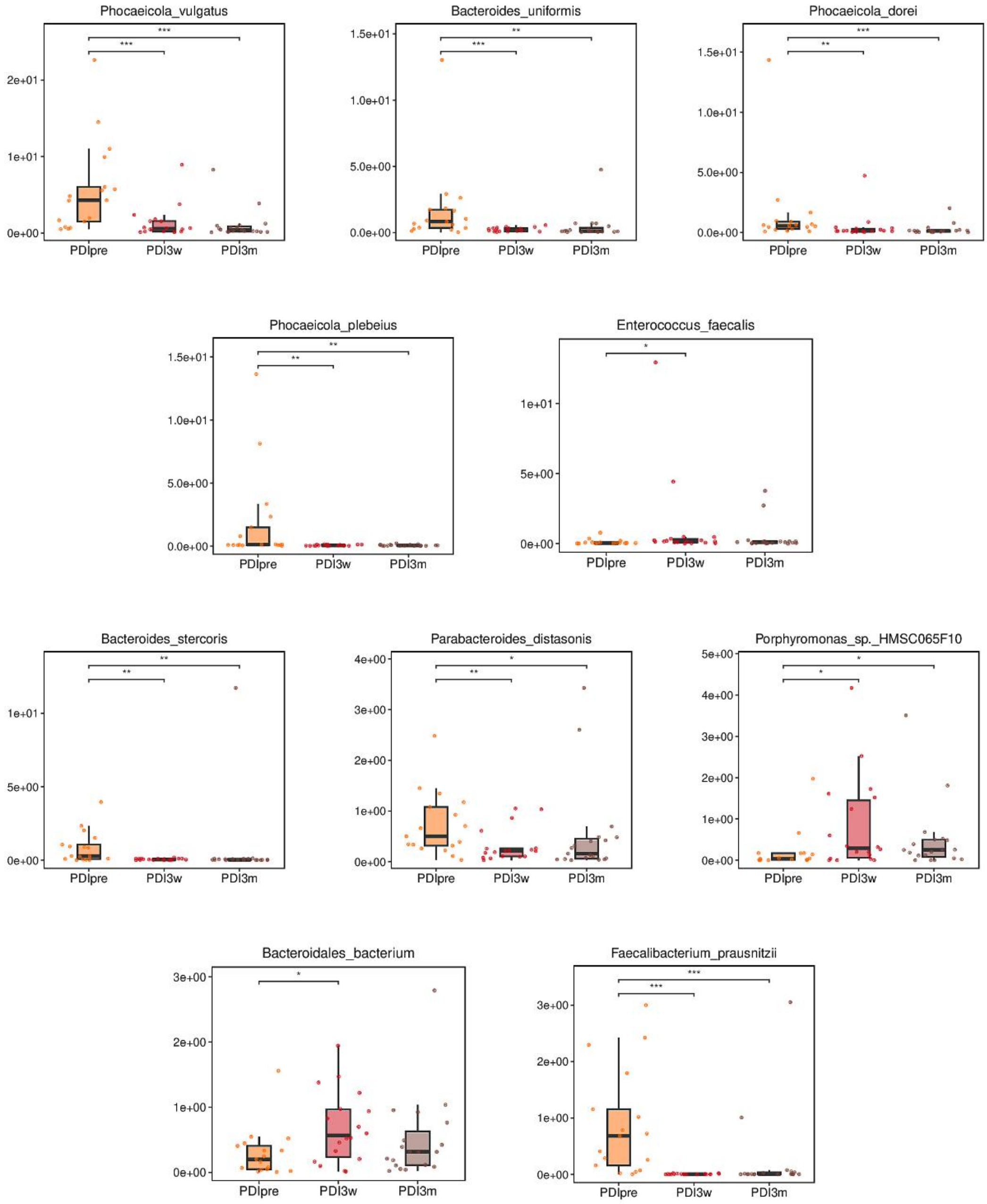

Figure S6. The top ten species with the highest abundance differences within the PDI group at preoperative, 3 weeks postoperatively, and 3 months postoperatively. Box plots show the median, interquartile range, and distribution. Statistical significance is indicated above the plots (*p < 0.05, **p < 0.01, ***p < 0.001). The vertical axis indicates the relative abundance values of species. Abbreviations: SDTpre: Samples collected 1day preoperatively from the stent-based diversion technique group. SDT3w: Samples collected 3 weeks postoperatively from the stent-based diversion technique group; SDT3m: Samples collected 3 months postoperatively from the stent-based diversion technique group.

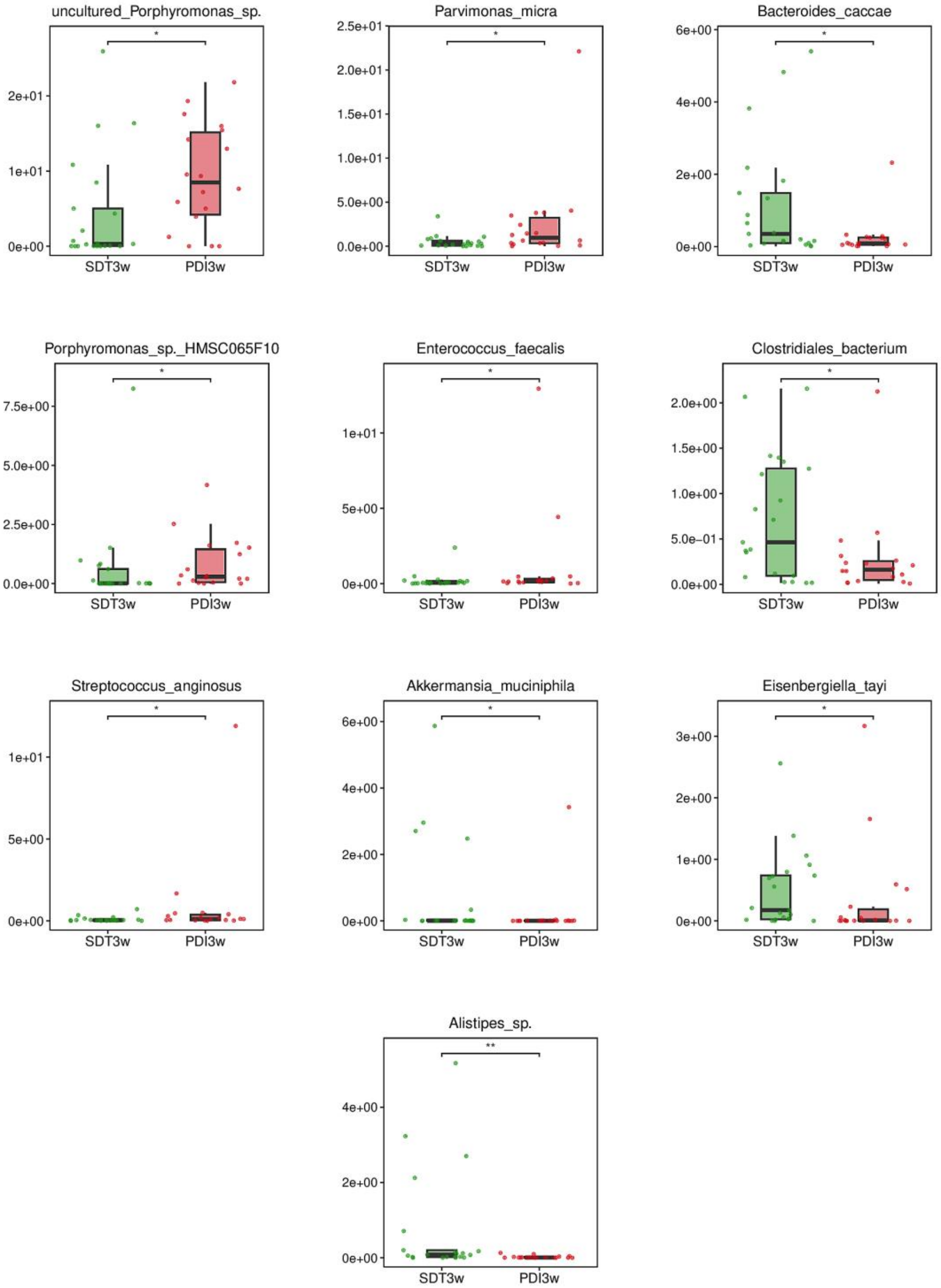

Figure S7. The top ten species with the highest abundance differences between the SDT and PDI group at 3 weeks postoperatively. Box plots show the median, interquartile range, and distribution. Statistical significance is indicated above the plots (*p < 0.05, **p < 0.01, ***p < 0.001). The vertical axis indicates the relative abundance values of species. Abbreviations: SDT3w: Samples collected 3 weeks postoperatively from the stent-based diversion technique group. PDI3w: Samples collected 3 weeks postoperatively from the prophylactic double-lumen ileostomy group.

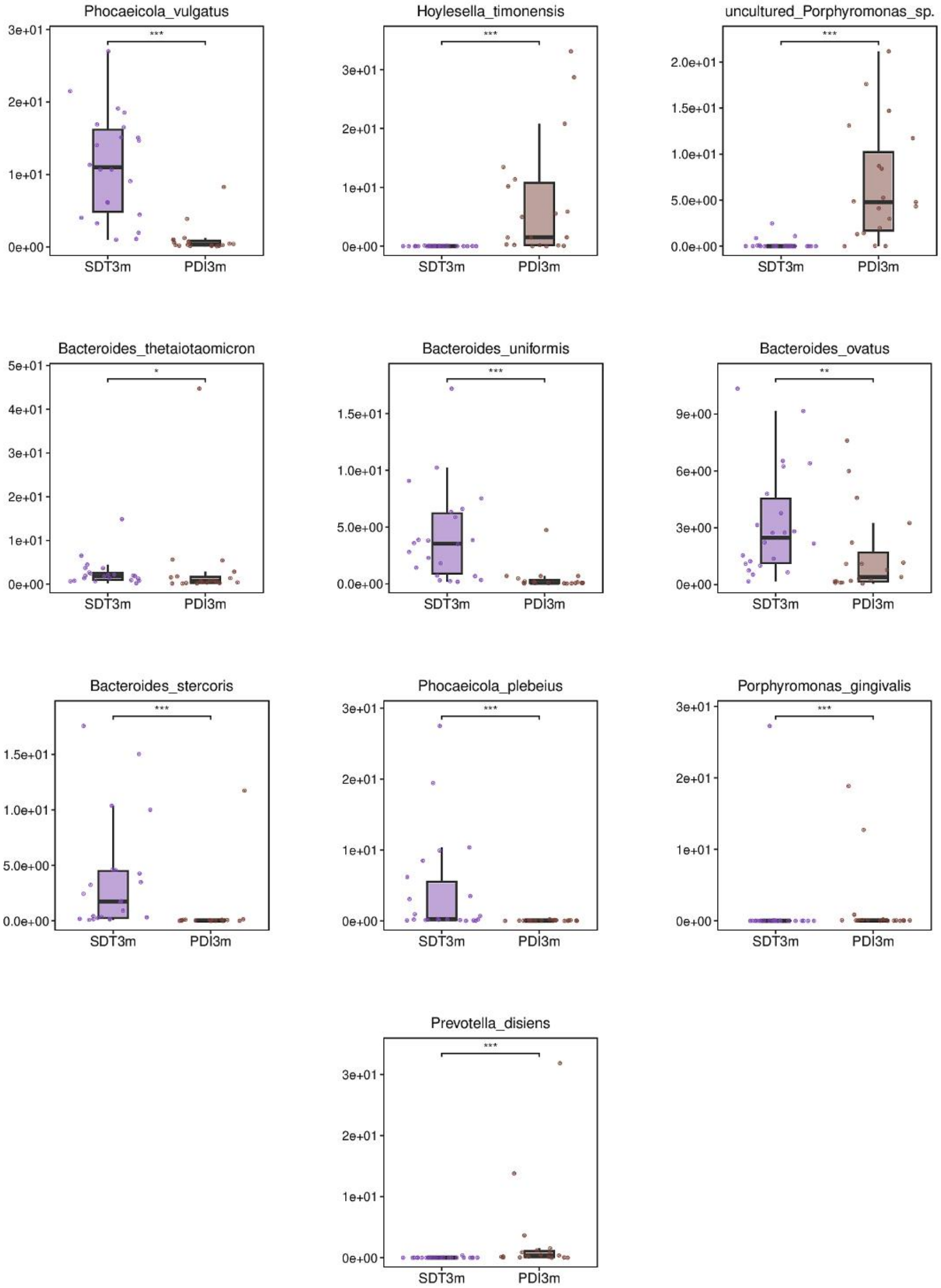

Figure S8. The top ten species with the highest abundance differences between the SDT and PDI group at 3 months postoperatively. Box plots show the median, interquartile range, and distribution. Statistical significance is indicated above the plots (*p < 0.05, **p < 0.01, ***p < 0.001). The vertical axis indicates the relative abundance values of species. Abbreviations: PDIpre: Samples collected 1 day preoperatively from the prophylactic double-lumen ileostomy group; PDI3w: Samples collected 3 weeks postoperatively from the prophylactic double-lumen ileostomy group; PDI3m: Samples collected 3 months postoperatively from the prophylactic double-lumen ileostomy group.

(a)

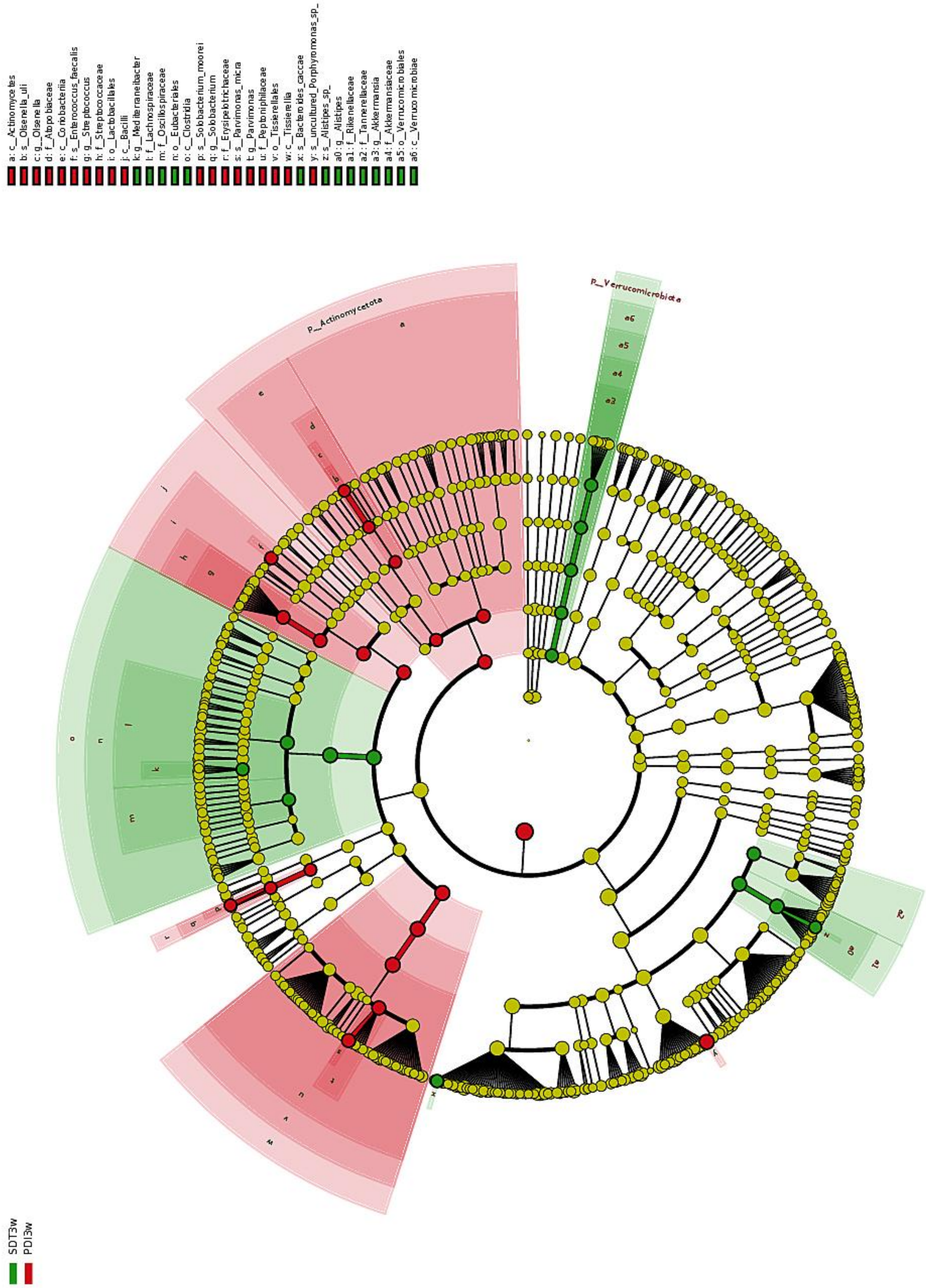

a: c__Actinomycetes
b: s__Olsenella_uli
c: g__Olsenella
d: f__Atopobiaceae
e: c__Coriobacteriia
f: s__Enterococcus_faecalis
g: g__Streptococcus
h: f__Streptococcaceae
i: o__Lactobacillales
j: c__Bacilli
k: g__Mediterraneibacter
l: f__Lachnospiraceae
m: f__Oscillospiraceae
n: o__Eubacteriales
o: c__Clostridia
p: s__Solobacterium_moorei
q: g__Solobacterium
r: f__Erysipelotrichaceae
s: s__Parvimonas_micra
t: g__Parvimonas
u: f__Peptoniphilaceae
v: o__Tissierellales
w: c__Tissierellia
x: s__Bacteroides_caccae
y: s__uncultured_Porphyromonas_sp_
z: s__Alistipes_sp_
a0: g__Alistipes
a1: f__Rikenellaceae
a2: f__Tannerellaceae
a3: g__Akkermansia
a4: f__Akkermansiaceae
a5: o__Verrucomicrobiales
a6: c__Verrucomicrobiae
P__Actinomycetota
P__Verrucomicrobiota
SDT3w
PDI3w

(b)

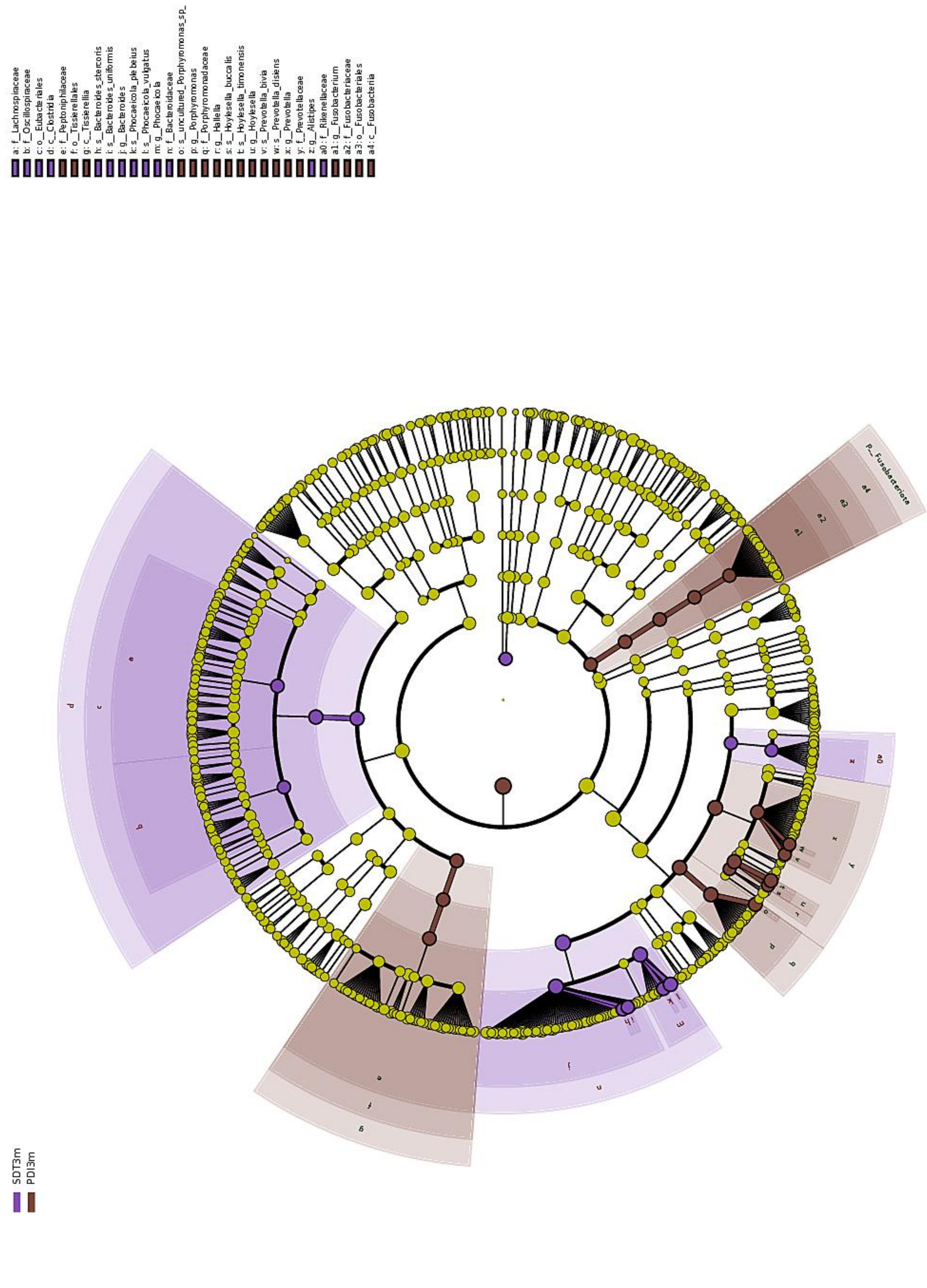
a: f__Lachnospiraceae
b: f__Oscillospiraceae
c: o__Eubacteriales
d: c__Clostridia
e: f__Peptoniphilaceae
f: o__Tissierellales
g: c__Tissierellia
h: s__Bacteroides_stercoris
i: s__Bacteroides_uniformis
j: g__Bacteroides
k: s__Phocaeicola_plebeius
l: s__Phocaeicola_vulgatus
m: g__Phocaeicola
n: f__Bacteroidaceae
o: s__uncultured_Porphyromonas_sp_
p: g__Porphyromonas
q: f__Porphyromonadaceae
r: g__Hallella
s: s__Hoylesella_buccalis
t: s__Hoylesella_timonensis
u: g__Hoylesella
v: s__Prevotella_bivia
w: s__Prevotella_disiens
x: g__Prevotella
y: f__Prevotellaceae
z: g__Alistipes
a0: f__Rikenellaceae
a1: g__Fusobacterium
a2: f__Fusobacteriaceae
a3: o__Fusobacteriales
a4: c__Fusobacteria
p__Fusobacteriota
SDT3m
PDI3m

Figure S9. Phylogenetic tree of microbial species at 3 weeks(a) and 3 months(b) postoperatively obtained through LEfSe analysis. Circles from inside to outside represent taxonomic levels from phylum to species. Red nodes indicate microbiota significantly enriched at 3 weeks postoperatively in the PDI group (PDI3w); blue nodes indicate microbiota significantly enriched at 3 weeks postoperatively in the SDT group (SDT3w); brown nodes indicate microbiota significantly enriched at 3 months postoperatively in the PDI group (PDI3m); purple nodes indicate microbiota significantly enriched at 3 months postoperatively in the SDT group (SDT3m); yellow nodes indicate no significant difference species. Node size is proportional to the LDA score, meaning larger nodes correspond to greater microbial contribution to distinguishing between the two groups. Pre-taxonomic hierarchy: p = phylum, c = class, o = order, f = family, g = genus, s = species. (Analysis threshold: LDA score> 3.5, $p < 0.05$).

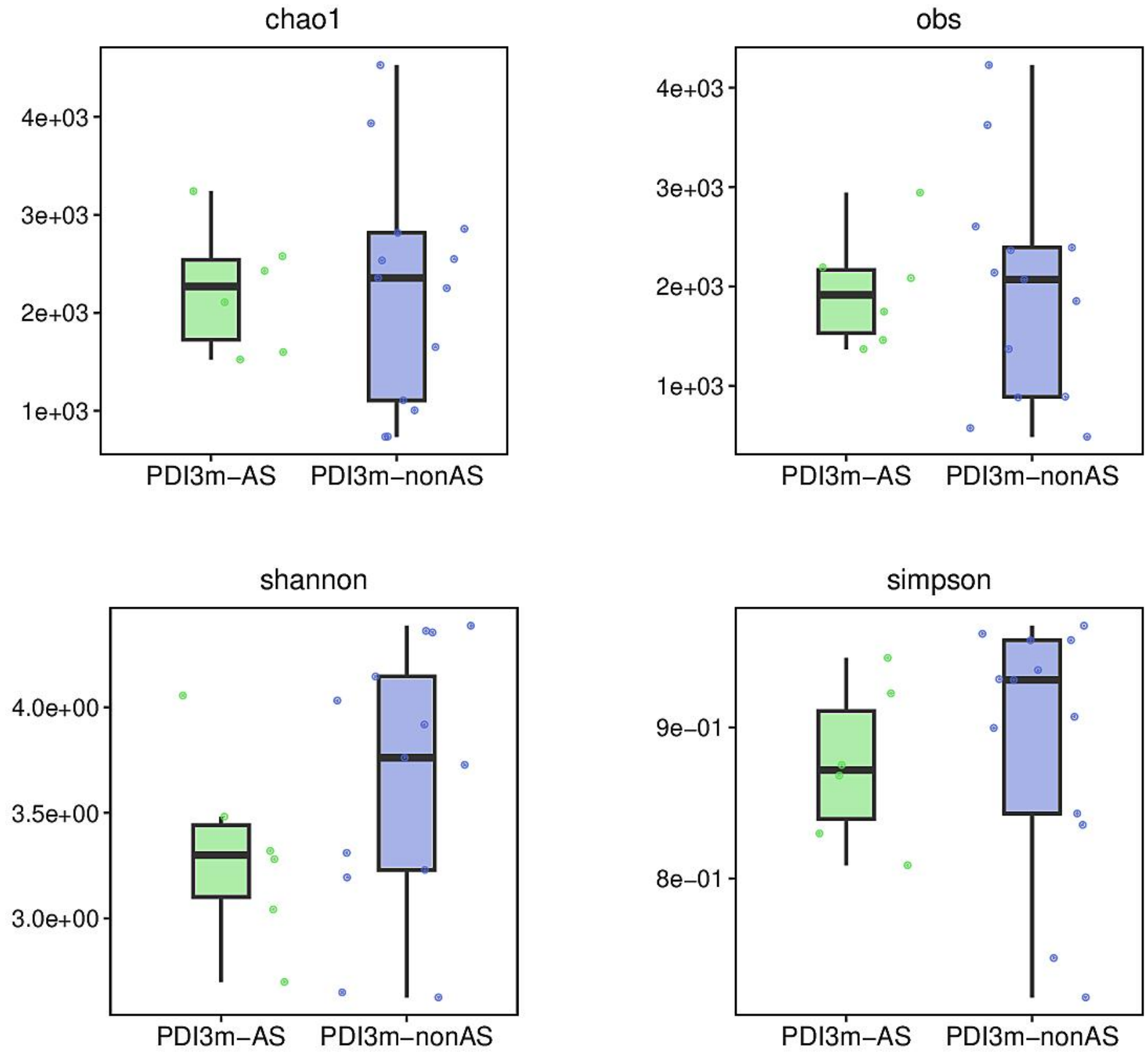

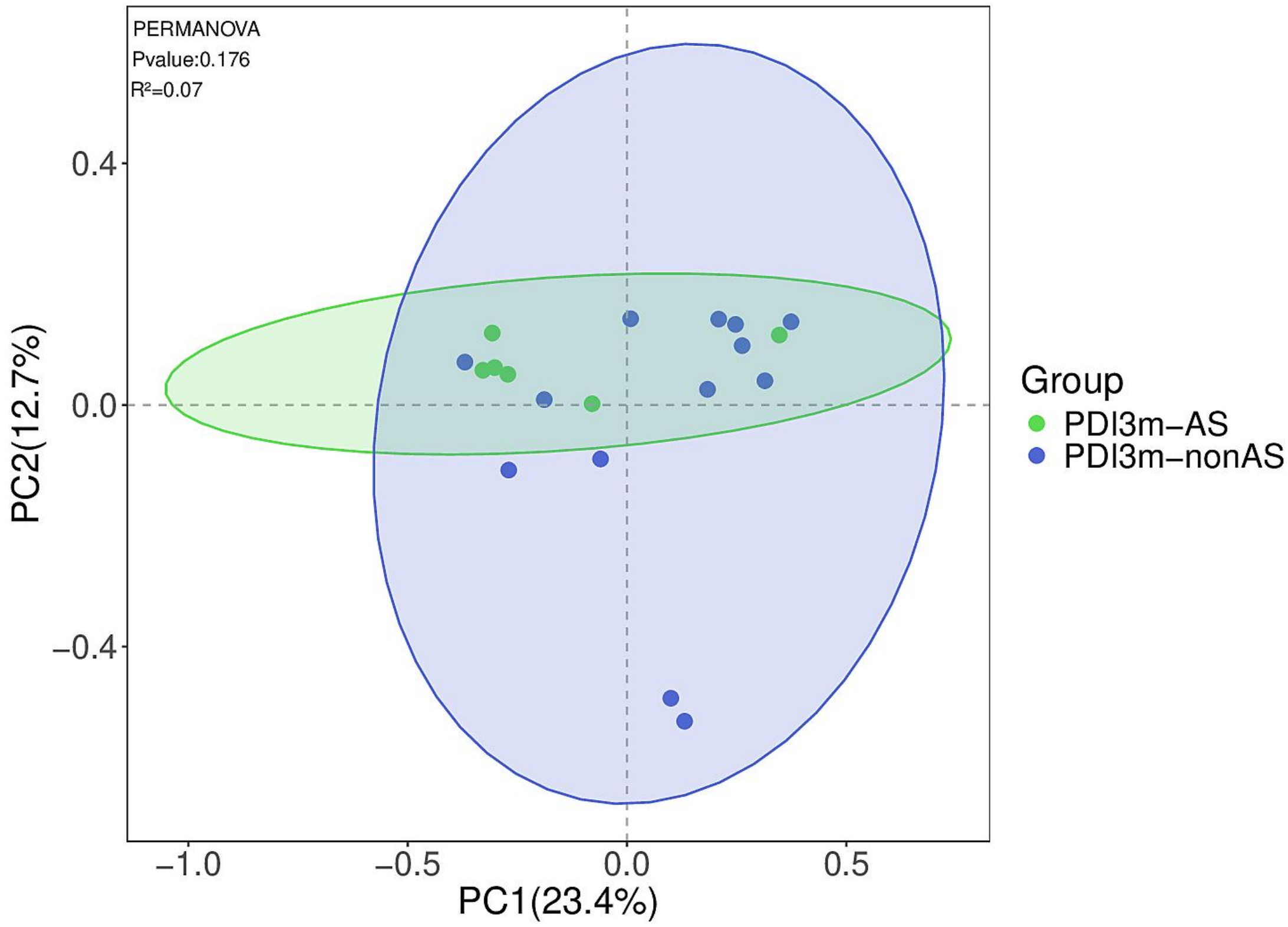

Figure S10. (a) α-diversity between the PDI3m-AS and PDI3m-nonAS groups. α-diversity indices (Simpson, Shannon, Obs, and Chao1) showed statistical differences, no significance was indicated. Box plots show the median, interquartile range, and data distribution. The vertical axis indicates the relative abundance values of species. Abbreviations: PDI3m-AS: Samples from patients with anastomotic stenosis at 3 months postoperatively in the PDI group. PDI3m-nonAS: Samples from patients without anastomotic stenosis at 3 months postoperatively in the PDI group. (b) β-diversity between the PDI3m-AS and PDI3m-nonAS groups. Principal coordinate analysis (PCoA) based on Bray - Curtis distance. Each dot represents one sample; percentages in parentheses indicate variance explained by each principal coordinate (PC). PERMANOVA revealed significant structural divergence between groups (R^2 = 0.07, p = 0.176). Abbreviations: PERMANOVA = Permutational multivariate analysis of variance.

Supplementary Table

Supplementary Table 1. Differential species between the SDT group and the PDI group identified by LEfSe analysis at 3 weeks postoperatively（LDA score > 3.5，$p < 0.05$）

species	Logarithm value	group	LDA score	*P* value
f__Rikenellaceae	4.7285642	SDT3w	4.3963703	0.0037115
g__Alistipes	4.7268361	SDT3w	4.3951047	0.0074435
c__Clostridia	4.9723875	SDT3w	4.3001285	0.0260428
o__Eubacteriales	4.9646935	SDT3w	4.2852569	0.0260428
f__Lachnospiraceae	4.5178706	SDT3w	4.0866824	0.0142481
f__Tannerellaceae	4.3450071	SDT3w	3.8468196	0.0486066
f__Oscillospiraceae	4.1723609	SDT3w	3.7760596	0.0023458
f__Akkermansiaceae	4.1202659	SDT3w	3.7718560	0.0023410
g__Akkermansia	4.1202659	SDT3w	3.7718560	0.0023410
o__Verrucomicrobiales	4.1202659	SDT3w	3.7718560	0.0023410
c__Verrucomicrobiae	4.1202659	SDT3w	3.7718560	0.0023410
p__Verrucomicrobiota	4.1202659	SDT3w	3.7718560	0.0023410
g__Mediterraneibacter	4.0509336	SDT3w	3.7099050	0.0112299
s__Bacteroides_caccae	4.0897506	SDT3w	3.6604462	0.0322694
*s__Alistipes_sp_*	3.8894981	SDT3w	3.5458180	0.0025737
*s__uncultured_Porphyromonas_sp_*	4.9860210	PDI3w	4.4457352	0.0346106
f__Peptoniphilaceae	4.9628046	PDI3w	4.3134902	0.0224943
o__Tissierellales	4.9628046	PDI3w	4.3134902	0.0224943
c__Tissierellia	4.9760946	PDI3w	4.2826479	0.0370953
k__Bacteria	5.9967451	PDI3w	4.2555568	0.0425225
g__Parvimonas	4.5076007	PDI3w	4.1330280	0.0131707
s__Parvimonas_micra	4.4345099	PDI3w	4.0593034	0.0166383
c__Bacilli	4.6019077	PDI3w	3.9693401	0.0179605
o__Lactobacillales	4.5292427	PDI3w	3.9342005	0.0425225
p__Actinomycetota	4.5307980	PDI3w	3.8229071	0.0074435
c__Actinomycetes	4.3520230	PDI3w	3.7557707	0.0208833
g__Streptococcus	4.3125804	PDI3w	3.7525213	0.0208833
f__Streptococcaceae	4.3125804	PDI3w	3.7525213	0.0208833
c__Coriobacteriia	4.0032772	PDI3w	3.5719648	0.0260428
f__Atopobiaceae	3.8520169	PDI3w	3.5680405	0.0224943
s__Enterococcus_faecalis	4.0689738	PDI3w	3.5536628	0.0397303
g__Solobacterium	3.8556548	PDI3w	3.5413847	0.0193738
f__Erysipelotrichaceae	3.8618684	PDI3w	3.5340066	0.0397303
s__Solobacterium_moorei	3.8272830	PDI3w	3.5154032	0.0062824
g__Olsenella	3.7751544	PDI3w	3.5066451	0.0483788
s__Olsenella_uli	3.7751544	PDI3w	3.5066451	0.0483788

Supplementary Table 2. Differential species between the SDT group and the PDI group identified by LEfSe analysis at 3 months postoperatively（LDA score > 3.5，$p < 0.05$）

species	Logarithm value	group	LDA score	*P* value
f__Bacteroidaceae	5.6951508	SDT3m	5.1918256	0.0001667
g__Phocaeicola	5.2968929	SDT3m	4.9412865	0.0000003
g__Bacteroides	5.4717170	SDT3m	4.8315907	0.0120780
s__Phocaeicola_vulgatus	5.0827799	SDT3m	4.7153340	0.0000003
s__Phocaeicola_plebeius	4.6527077	SDT3m	4.3499011	0.0000288
o__Eubacteriales	5.0466405	SDT3m	4.3368825	0.0280830
c__Clostridia	5.0503598	SDT3m	4.3314522	0.0262651
s__Bacteroides_uniformis	4.6600007	SDT3m	4.3153167	0.0000042
s__Bacteroides_stercoris	4.6032446	SDT3m	4.2591436	0.0000015
f__Oscillospiraceae	4.5904002	SDT3m	4.1925599	0.0000879
f__Rikenellaceae	4.5547831	SDT3m	4.1650320	0.0000507
g__Alistipes	4.5485102	SDT3m	4.1586623	0.0000633
f__Lachnospiraceae	4.7034050	SDT3m	4.0733029	0.0022216
k__Viruses	4.4372080	SDT3m	4.0577831	0.0000112
f__Prevotellaceae	5.3719135	PDI3m	4.9375014	0.0001667
g__Porphyromonas	5.1691672	PDI3m	4.8346543	0.0000160
f__Porphyromonadaceae	5.1694899	PDI3m	4.8326108	0.0000361
g__Hoylesella	5.0260617	PDI3m	4.7000322	0.0000112
g__Prevotella	4.9923291	PDI3m	4.6126749	0.0000980
*s__uncultured_Porphyromonas_sp_*	4.8471167	PDI3m	4.5535656	0.0000042
s__Hoylesella_timonensis	4.8838985	PDI3m	4.5532484	0.0000017
c__Tissierellia	4.8080098	PDI3m	4.2812882	0.0000879
f__Fusobacteriaceae	4.6801192	PDI3m	4.2319058	0.0320460
p__Fusobacteriota	4.6801192	PDI3m	4.2319058	0.0320460
g__Fusobacterium	4.6801192	PDI3m	4.2319058	0.0320460
o__Fusobacteriales	4.6801192	PDI3m	4.2319058	0.0320460
c__Fusobacteriia	4.6801192	PDI3m	4.2319058	0.0320460
f__Peptoniphilaceae	4.7725512	PDI3m	4.2295959	0.0000980
o__Tissierellales	4.7725512	PDI3m	4.2295959	0.0000980
s__Hoylesella_buccalis	4.4664075	PDI3m	4.1557237	0.0000022
s__Prevotella_disiens	4.4852100	PDI3m	4.1510665	0.0000112
s__Prevotella_bivia	4.4190134	PDI3m	4.0940131	0.0000405
k__Bacteria	5.9971862	PDI3m	4.0623641	0.0000126
g__Hallella	4.3586710	PDI3m	4.0211972	0.0000007

Supplementary Table 3. Differential KEGG pathway between the SDT group and the PDI group identified by LEfSe analysis at 3 weeks postoperatively（LDA score > 2, $p < 0.05$）

KEGG level3 pathway	Logarithm value	Groups	LDA value	P value
Biosynthesis of amino acids (ko01230)	4.4280497	SDT3w	3.0732289	0.0279918
2-Oxocarboxylic acid metabolism (ko01210)	3.8481128	SDT3w	2.7709377	0.0154024
Valine, leucine and isoleucine biosynthesis (ko00290)	3.3147710	SDT3w	2.5551099	0.0260428
Alanine, aspartate and glutamate metabolism (ko00250)	3.9201152	SDT3w	2.3066252	0.0080934
Histidine metabolism (ko00340)	3.5007451	SDT3w	2.2883123	0.0025750
Bacterial chemotaxis (ko02030)	3.0058493	SDT3w	2.2534387	0.0166383
Pantothenate and CoA biosynthesis (ko00770)	3.5842754	SDT3w	2.1728368	0.0322694
Cell cycle (ko04110)	0.0000000	SDT3w	2.1176803	0.0291084
Arginine and proline metabolism (ko00330)	3.4622225	SDT3w	2.0586337	0.0346106
MicroRNAs in cancer (ko05206)	3.0139858	SDT3w	2.0523326	0.0486066
Nucleocytoplasmic transport (ko03013)	0.0000000	SDT3w	2.0066670	0.0291084
HIF-1 signaling pathway (ko04066)	3.3590471	PDI3w	2.2209607	0.0154024

Supplementary Table 4. Differential KEGG pathway between the SDT group and the PDI group identified by LEfSe analysis at 3 months postoperatively（LDA score > 2, $p < 0.05$）

KEGG level2 pathway	Logarithm value	Groups	LDA value	P value
Carbohydrate metabolism	5.0042991	SDT3m	3.7275780	0.0000405
Signal transduction	4.5504249	SDT3m	3.4735738	0.0024234
Amino acid metabolism	4.7553449	SDT3m	3.3049226	0.0009871
Biosynthesis of other secondary metabolites	4.2680718	SDT3m	3.2286521	0.0011874
Lipid metabolism	4.3335243	SDT3m	3.1603925	0.0002790
Transport and catabolism	3.9185036	SDT3m	2.9226861	0.0139881
Drug resistance: antimicrobial	4.2046347	SDT3m	2.8885088	0.0009871
Nervous system	3.3660599	SDT3m	2.4446986	0.0017050
Environmental adaptation	3.5250453	SDT3m	2.3226390	0.0002275
Cellular community - eukaryotes	0.2759506	SDT3m	2.2999222	0.0001172
Translation	4.4382321	PDI3m	3.5492511	0.0000879
Energy metabolism	4.5772766	PDI3m	3.3862441	0.0055833
Replication and repair	4.4965549	PDI3m	3.3359128	0.0009871
Nucleotide metabolism	4.4394349	PDI3m	3.3318530	0.0007446
Metabolism of cofactors and vitamins	4.6236499	PDI3m	3.2914701	0.0001850
Membrane transport	4.4532905	PDI3m	3.2494257	0.0130020
Metabolism of terpenoids and polyketides	3.9810249	PDI3m	2.8977845	0.0000203
Folding, sorting and degradation	4.1326292	PDI3m	2.8284351	0.0076595

Neurodegenerative disease	3.5573831	PDI3m	2.7820168	0.0000054
Cell growth and death	3.9319359	PDI3m	2.6707105	0.0065477
Transcription	3.3098328	PDI3m	2.5391727	0.0055833
Aging	3.5646230	PDI3m	2.4326455	0.0009871
Drug resistance: antineoplastic	3.2964051	PDI3m	2.3256143	0.0015590

Note: LDA score (linear discriminant analysis effect size) represents the contribution of each feature to inter-group differences, with higher scores indicating a more significant role in discriminating between the two groups. The p value indicates the statistical significance of the difference between groups, and $p < 0.05$ was considered statistically significant. Both LDA scores and p values are presented with 7 decimal places.
